# Ambient Intelligence in the Living Room

**DOI:** 10.3390/s19225011

**Published:** 2019-11-16

**Authors:** Asterios Leonidis, Maria Korozi, Vassilis Kouroumalis, Evangelos Poutouris, Evropi Stefanidi, Dimitrios Arampatzis, Eirini Sykianaki, Nikolaos Anyfantis, Evangelos Kalligiannakis, Vassilis C. Nicodemou, Zinovia Stefanidi, Emmanouil Adamakis, Nikos Stivaktakis, Theodoros Evdaimon, Margherita Antona

**Affiliations:** Institute of Computer Science (ICS), Foundation for Research and Technology—Hellas (FORTH), 70013 Heraklion, Greece; korozi@ics.forth.gr (M.K.); vic@ics.forth.gr (V.K.); poutouris@ics.forth.gr (E.P.); evropi@ics.forth.gr (E.S.); arabatzis@ics.forth.gr (D.A.); eirinisi@ics.forth.gr (E.S.); nanifant@ics.forth.gr (N.A.); vaggeliskls@ics.forth.gr (E.K.); nikodim@ics.forth.gr (V.C.N.); zinastef@ics.forth.gr (Z.S.); madamakis@ics.forth.gr (E.A.); nstivaktak@ics.forth.gr (N.S.); evdemon@ics.forth.gr (T.E.); antona@ics.forth.gr (M.A.)

**Keywords:** intelligent environment, smart home, intelligent living room, ambient intelligence, intelligent artefacts, multimodal interaction

## Abstract

The emergence of the Ambient Intelligence (AmI) paradigm and the proliferation of Internet of Things (IoT) devices and services unveiled new potentials for the domain of domestic living, where the line between “the computer” and the (intelligent) environment becomes altogether invisible. Particularly, the residents of a house can use the living room not only as a traditional social and individual space where many activities take place, but also as a smart ecosystem that (a) enhances leisure activities by providing a rich suite of entertainment applications, (b) implements a home control middleware, (c) acts as an intervention host that is able to display appropriate content when the users need help or support, (d) behaves as an intelligent agent that communicates with the users in a natural manner and assists them throughout their daily activities, (e) presents a notification hub that provides personalized alerts according to contextual information, and (f) becomes an intermediary communication center for the family. This paper (i) describes how the “Intelligent Living Room” realizes these newly emerged roles, (ii) presents the process that was followed in order to design the living room environment, (iii) introduces the hardware and software facilities that were developed in order to improve quality of life, and (iv) reports the findings of various evaluation experiments conducted to assess the overall User Experience (UX).

## 1. Introduction

Today, we are witnessing the rise of a new paradigm [1] of information and communication technologies (ICT), spearheaded by smartphones and tablets revolutionizing mobility [2] and delivering easy-to-use touch interaction, the ever-growing Internet of Things (IoT), augmented reality, and wearables. This new paradigm is enhanced with the emergence of Ambient Intelligence (AmI), where the environment is aware of users and can intelligently respond and adapt to their needs [3]. Currently, it is still debated whether and how all the above-mentioned technologies will be integrated in a seamless environment [4,5], but it is almost certain that these changes will affect all areas where interaction takes place, including the home [6,7,8]. In this article, we will focus on the living room and how it will be transformed in light of the new interaction paradigms. 

The term “living room” appears in literature roughly at the end of the 19th century. It replaced the formal room for social events and became a room that reflected the taste and personality of the owner [9]. It featured things such as rugs, tables, a sofa, curtains, and often a fireplace [10]. With the advance of technology, it eventually became the room with the TV set, coffee table, and the sofa, which is a combination that lends itself to sociological studies, where TV viewing patterns reflect the daily routines and relationships of the house residents [11]. A typical activity is watching TV, which can also be regarded as a social and collective activity, influenced by the domestic context; children turning on the TV during or after dinner, parents listening to the news, and a spouse watching their favorite soap opera are all scenarios that can and have been analyzed for their sociological implications [12]. Furthermore, the living room sofa is not just a comfortable seat to watch TV. It has been identified as an “activity center” [13] where inhabitants eat, drink, work, relax, use social media, or paint their nails [9]. Since the advent of mobile, always-connected technologies, such as tablets and smartphones, the activities and the way they are carried out have now evolved even further. There are already a lot of studies about second screen usage and how they complement the TV viewing experience, but also how they shape the dynamics and the relationships between home residents [14].

The TV itself is no longer a straightforward broadcaster of media, but also a device connected to the Internet, offering online interactive media, over-the-top content, on-demand streaming media, and home networking [15]. It is expected to serve as an information storage device, a visualization medium, an interaction point, a data source, and a data processor [16]. Given that the TV is currently the biggest Input–Output (I/O) device available in almost any environment (e.g., homes, hotels, offices, public spaces) [17], it is surprising that there are very few attempts to use it as the main tool to monitor, manage, and interact with intelligent home services. Approaching the living room from the viewpoint of interaction designers and developers, we believe that the TV and the accompanying table and sofa, along with the surrounding walls, offer exciting new opportunities; using sensor networks, artificial intelligence, multimedia, pervasive and mobile computing, middleware, and infrastructure based on microservices, we can transform the living room into a multimodal, intelligent, and versatile interaction hub with the ambient facilities of the entire environment. 

This article investigates the emerging roles of the living room in the era of Ambient Intelligence, given that it incorporates diverse intelligent artefacts (e.g., smart TVs, technologically-augmented furniture, smart lights) and services (e.g., video on demand, messaging platforms, user profiling). In particular, it outlines the design process toward building such a technology-rich environment and introduces the hardware and software facilities that were developed in order to improve the quality of life of the home inhabitants. Additionally, it presents the findings of a series of user-based evaluation experiments that assessed the functionality and utility of the developed ambient applications and their overall User Experience (UX). Finally, it reports our consolidated experience and insights as they were acquired during the entire development process of the “Intelligent Living Room”, in terms of design, interaction, and functionality.

## 2. Related Work

### 2.1. Augmenting the Living Room

The advancement of Internet of Things (IoT) [18], in combination with cloud computing [19], has led to an abundance of web-enabled devices and services for intelligent homes [20] that transformed the face of domestic life. In such technologically advanced homes, individual users in their everyday lives interact frequently with various smart artefacts in conjunction. According to [21], the main objectives of smart homes are the automation of tasks that might be complex or tedious for inhabitants (e.g., control home appliances), improving comfort, saving energy, enhancing the feeling of security, and supporting independent living for elderly or people with disabilities. 

To that end, the environment of the living room has gained much attention by researchers, since inhabitants spend a considerate amount of time there with their family and friends, and as a result many daily activities are linked to it (i.e., watching TV, reading, socializing, relaxing) [22]. Starting from the early 2000s, the EasyLiving project [23] presented a technological architecture intended for smart environments that provides user detection, identification and tracking, device tracking, and several demo applications regarding room controlling, media control, remote user sessions, and a universal mouse controller. Illumiroom [24] and RoomAlive [25] applied spatial augmented reality techniques by employing depth cameras and projectors in order to perform projection mapping in the room’s space and physical objects, thus enabling new interaction possibilities and turning the living room into an enhanced and immersive experience. Moreover, services and applications adapt in a context-sensitive manner [21], while interaction goes beyond traditional desktop-oriented interaction techniques (e.g., mouse, keyboard, and touch), endorsing natural modalities such as eye-tracking [26], freehand gestures [27], etc.

### 2.2. The Interactive Television

The most common device found in almost every living room is the television, while watching TV is a regular daily activity [28] that takes up most of people’s leisure time [29]. In some cases, people consider the TV as a companion, letting it play in the background regardless of their activity [30]. There are people that even talk to this device, although they know that they will never get an answer [31]. Nowadays, as Internet technology and satellite broadcasting change the way people consume television content, the medium continues to evolve, solidifying its position as one of the most important inventions of the 20th century [32]. Over the years, the traditional TV set has significantly evolved, changing the type of interaction between the users and the medium itself. In fact, as a result of the “digital revolution”, the TV is now undergoing a process of radical change. New devices (e.g., ports to connect external USB devices, cable or satellite receiver, Local Area Network (LAN) or Wi-Fi broadband connections) and controllers (e.g., different kinds of remote controls, gesture recognition systems, smartphone connectivity) are attached and integrated to televisions, leading to the extensive adoption of Smart TVs. Currently, Smart TV devices mostly focus on delivering rich User Interfaces (UIs) that aim to improve the User Experience (UX) while consuming streaming media and web content or interacting with social networks [33].

The surveys in [34,35] reveal six principal areas of interest regarding the services that an interactive TV (iTV) should offer: (i) local information (e.g., weather, traffic, local events), (ii) personalized information (e.g., recommendation of content, reminders, video on demand), (iii) auxiliary context-sensitive information (e.g., Electronic Program Guide or EPG, Enhanced TV, product information), (iv) participative, collaborative services (e.g., citizen participation, community activities, exchange of ideas or experiences), (v) communication services (e.g., instant messaging, short messages, greeting services), and (vi) standalone services (e.g., Internet access, interactive games, music/videos download, ordering services). Although the results indicated that iTVs would be adopted mostly by the elderly, younger audiences still remain among its major user groups [36]. 

The aforementioned services deliver rich multimedia content (i.e., movies, TV shows, music) and interactive applications (e.g., games) to their users via third-party mediating tools known as media centers. A media center is either: (i) audio-visual software, such as Netflix [37], Hulu [38], Kodi [39], and Media Portal [40], that is directly accessible through the TV, or (ii) a separate device (i.e., set-top box), such as Apple TV [41], Google Chromecast [42], Amazon Fire TV [43], and Roku [44], that incorporates the necessary software and hardware (e.g., internal storage, network interface, remote control) components to facilitate access. To further increase their perceived usefulness, media centers have extended their interaction modalities beyond the traditional remote controller, by integrating speech, touch, and gestures. These multimodal facilities have widened the user base of media centers [45] by permitting users with disabilities to interact with their advanced features beyond simple TV content consumption using their preferred interaction modality [46]. Besides multimodal interaction, multimedia content is also tailored to the preferences of each user to address the continuously growing number of data sources that hinder manual discovery and selection (i.e., recommendation systems [47] of highly-rated internet sites such as Amazon, YouTube, Netflix). Finally, the emergence of AmI and the abundance of smart and IoT-enabled devices in domestic environments [35,48] have transformed houses into Smart Homes [49] with user-defined behavior [50], and as a result, the role of TV has been upgraded. Nowadays, it is considered a centralized control center [51,52] through which users can easily monitor and manage their various devices via personalized [53] and context-aware [54] UIs that permit their shared use by multiple residents (i.e., family members, household employees, guests).

### 2.3. Second Screens

Besides the television device, which is considered as the main display of an “intelligent” living room, additional commercial artefacts (e.g., smartphones, tables, laptops) or other flat surfaces on which information can be projected/displayed (e.g., coffee table, wall) act as second screens that enrich TV content [55,56] or give additional feedback to the user [24,57]. As research suggests [58,59], the TV no longer demands our full attention. Instead, while watching TV, consumers are conducting complementary activities (e.g., looking for information) across multiple screens, usually through the device that is closest to them. In addition to such exploration tasks, there are studies [60,61] claiming that the usage of second screens provides enhanced attention to TV programs, encourages critical thinking, fosters co-discussion among users regarding TV news and social media, and permits personalized advertising [62]. Additionally, as reported in [63,64], the incorporation of gamification techniques (e.g., scoring systems where the user earns points for being an active commentator) can increase participation via socially generated commentary. In [65], the authors created a companion app for multi-episode series that creates story arcs. Their approach is focused specifically on TV series and uses the second screen to support the contents of the main screen with supplementary commentary and character progression. However, there is no bidirectional interaction between the companion app and the main device. The work in [66] presents a spatially aware mobile and wall display visualization technique; based on the findings of an evaluation study, the authors highlight the benefits of distributed interaction among combined displays, such as mobile and wall displays. In [67], the authors developed a multi-screen cloud social TV system that consists of a video watching application which supports local and remote viewers, as well as “video teleportation” functionality between the devices present in a living room. In addition to content presentation, second screens are also intended to be used as input devices through which users actively interact and engage with the viewing ecosystem [34], thus promoting the bidirectional interactive TV (iTV) concept [68,69]. In particular, many approaches use second screens as hosts that contain interfaces that not only display information but also remotely control the TV and the overall viewing environment [59,70,71,72].

While second screening has benefits [73], it also has its fair share of pitfalls. Cognitive fatigue is probably the most important one, especially after extensive use [74], whereas problems concerning decision making and usability issues come next [75,76]. In order to be effectively used, second screening should mainly aid the user visually by showing complementary content and not replace entirely the main screen [77], especially when the user is working on a cognitively demanding primary task [78]. Moreover, the TV should remain the most significant medium in a viewing ecology, and the user should always be in control of it [79]. Context-sensitive prompts can motivate users to actively participate [80], and the interface should always be optimized for the target screen [81] and personalized for the current user [82].

### 2.4. Surface Computing

While second screens hosted in commercial devices, such as smartphones and tablets, inherently support user input (i.e., touch), common surfaces such as a coffee table or a wall can be transformed into interactive mediums with the use of ICT. Such technologically enhanced artefacts follow the paradigm of surface computing [4] and grant multimodal interaction (i.e., touch and object recognition) via unobtrusive sensing technologies. The terms surface computing and surface computers [4] were coined by Microsoft in the PixelSense project [83] and describe means of (multitouch) interaction based on common physical surfaces. Since then, in addition to research prototypes [84,85], many vendors have commercialized such devices [86,87,88]. 

From an interaction perspective, as [17] reports, such systems (e.g., table top or wall-projected interfaces) present design challenges because interaction resembles more real-world object manipulation than traditional mouse-based interaction in computers. Different manipulation styles have their own pros and cons; specifically, as [89] suggests, direct input enables rich interpersonal and natural interactions, allowing users to understand their actions (especially during multi-user setups), while indirect input through mouse devices allows a more comfortable and ergonomic interaction, enabling easy access to all regions of the surface. Finally, when multitouch manipulation is supported, (i) the touch interaction paradigm should support some level of personalization/customization [90] and (ii) the role of multitouch surfaces along with appropriate vocabularies of gestures have to be defined for each application [91]. 

Visualization is another challenging aspect of surface computing, due to the occlusion problems that occur when a physical object is placed on top of a UI element; to that end, various research approaches aim to adapt the overall interface so as to eliminate such cases. The work in [92] employs a polar-coordinate system and fisheye view in order to support collaborative interaction on a circular table top interface that give users the full capability to relocate, re-orient, scale, and layout the documents in the circular interface, as well as support users’ focus during collaborative group meetings around the table. SnapRail [93] recognizes the occluding physical object’s footprint and rearranges the occluded virtual elements over a ring-shaped rail widget that appears around the object. Finally, aiming to address the overall occlusion problem that takes place on heavily cluttered table tops, [94] introduces an access-supporting occlusion management algorithm that identifies the visible regions of the display suitable for showing digital content. 

Despite these challenges, many applications can be found in the literature that deliver surface-oriented applications. MirageTable [95] is an augmented reality curved table that provides virtual 3D model creation, interactive gaming with real and virtual objects, and a 3D teleconferencing experience by enabling real-time stereoscopic 3D digitization of the user sitting in front of the table along with the physical items that are present on its surface. Kirk et al. [96] report the findings of a field study regarding an augmented surface for families that enabled the management (e.g., viewing, archiving) of digital content such as photos; people regardless of their age were very much engaged with the table top system paradigm because of the open nature of interaction, which supported multitouch and the integration of physics principles (e.g., photos pushing each other when colliding). Drift Table [97] is a coffee table that aims to support ludic activities in the home, such as, for example, geographical landscape viewing (e.g., explore the countryside, travel to a friend’s house). It offers a small viewport showing a slowly changing aerial view of the British landscape, while shifting weights on the table changes its apparent height, direction, and speed. Amongst others, its authors found that ludic activities increase social engagement and are usually interleaved with everyday utilitarian ones. The work in [98] enables interfacing with a TV set using low-effort gestures detected (using computer vision) over a predefined area of the surface of a coffee table. Lastly, FingerTalk [99], while studying the collaborative decision making of users interacting cooperatively with a touch-enabled table surface, showed that interactive tables show much promise for supporting flexible and fluid ways of creating and discussing digital documents.

### 2.5. Summary

As the presented literature review confirms, there are various approaches that use the TV as the primary I/O medium of the intelligent environment; some of them adopt second screening techniques that allow the audience to interact with the content they are consuming, while others introduce surface computing into the intelligent environment in order to improve the user experience and introduce novel interaction techniques. In summary, some of these approaches could be adopted in the overall context of intelligent environments in order to: (i) enhance the content of the main screen, (ii) simplify interaction by applying multiple modalities, (iii) exploit contextual information, and (iv) personalize content delivery based on users’ preferences. To the best of our knowledge, an intelligent environment that combines all these functionalities does not exist. Hence, the “Intelligent Living Room” reported in this article constitutes a holistic approach towards formulating a smart ecosystem aiming to: (a) enhance leisure activities by providing a rich suite of entertainment applications, (b) implement a home control middleware, (c) act as an intervention host that is able to contribute in illustrating appropriate content when the users need help or support, (d) behave as an intelligent agent that communicates with the users in a natural manner and assists them throughout their daily activities, (e) present a notification hub that provides personalized alerts according to contextual information, and (f) become an intermediary communication center for the occupants (e.g., family).

## 3. Design Process

### 3.1. Methodology

Designing an entire room within a complex environment such as an “Intelligent Home” is not a straightforward process; contrariwise, there are multiple issues emerging while designing pervasive applications for Intelligent Environments [100,101] (e.g., specific requirements of an intelligent application usually affect the design of the environment itself, and an exponential complication of the context of use). Much work has been done for the software engineering part of the development, and the literature has quite a few examples of attempts to tackle specific parts of the process, such as requirements elicitation or definition [102,103,104,105,106]. Given that Intelligent Environments inherently aim to support their users, the user should be the focus of attention when designing such applications. Consequently, since user-centered design (UCD) principles should apply in any situation involving interactive systems [107], such an iterative UCD process has been followed for every intelligent application built in the context of the “Intelligent Living Room”. 

The process followed while designing the “Intelligent Living Room” was based on the Design Thinking methodology [108] (i.e., Empathize, Define, Ideate, Prototype, Test). In a series of meetings with the development team (e.g., analysts, designers, interior designers, programmers) and several potential end users (male and female users with no disabilities aged between 20 and 45), scenarios and personas (Section 3.2) were selected for the “Empathize” and “Define” steps of Design Thinking. As a subsequent step, multiple brainstorming sessions were organized for the “Ideate” part of the process. The brainstorming sessions began with an introduction making two specific points: (i) to express any idea, no matter how expensive or difficult or even unrealistic it sounded, and (ii) to focus solely on the interaction aspects by hypothetically considering all ethical, privacy, and security issues resolved (even if this is definitely not the case, as recent literature suggests [109,110]). Dozens of ideas were produced, which were then filtered through interviews with domain experts (e.g., computer vision specialists, industrial engineers, experts in automation and robotics, architects, interior designers). This process resulted in the identification and exclusion of the (currently) unfeasible ideas; for example, a lot of the ideas involved advanced robotics and complex object recognition. Experienced interaction designers and developers also reviewed the ideas and offered valuable insight and comments, as well as preferences in regard to which ideas had the most potential in their opinion in terms of innovation, research interest, and higher possibilities to be accepted by end users (e.g., ideas that sounded attractive/cool/fun). In the end, a set of ideas for the living room were generated; they were enriched with a description, potential challenges, noteworthy comments by domain experts if any, and related services.

**Figure 1:**
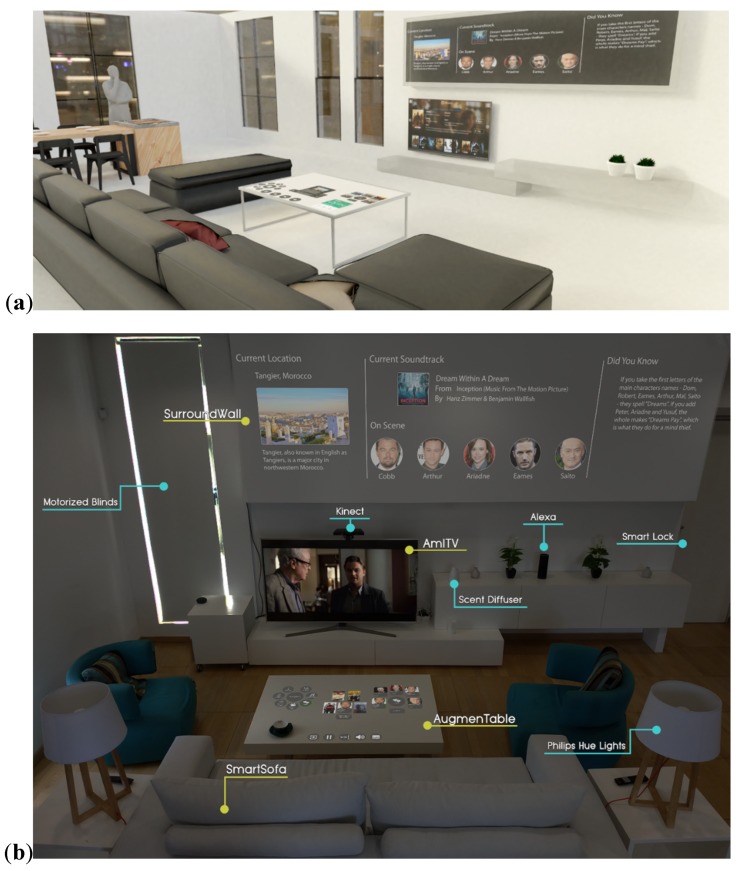
(**a**) The 3D representation of the living room as it resulted from the prototyping phase; (**b**) The current “Intelligent Living Room” setup.

The prototyping phase started by creating a realistic 3D representation of the room (Figure 1a). In accordance with the practice followed in similar use cases [111], such a prototype would be of outmost importance as it can assist designers in selecting the best solution by encouraging reflection in design, permitting the exploration of design ideas, and imagining the ramifications of design decisions. Given that the available space was empty (with no furniture), there was the opportunity to conceptualize different alternatives to better fulfil the requirements selected from the previous phases. In more details, UX experts along with specialists from other relevant fields (computer vision, industrial design, automation and robotics, architecture, interior design) collaborated during focus group meetings in order to design the model of the “Intelligent Living Room”, while alternative designs were evaluated via numerous methods (e.g., computer simulations, role playing a scene, interviewing potential end users or experts). Apart from the type and location of the furniture, the model presented the placement of the technological equipment and custom-made artefacts, as well as the modifications of the traditional furniture (e.g., the exact position of the sensors inside the sofa). After examining the final model of the living room, UI and UX experts undertook the task of prototyping the most promising applications (resulting from the ideation phase), among which are the ones presented in the remainder of this article. 

During that phase, the challenge of quickly evaluating low- or high-fidelity design mockups soon emerged. Since we were no longer designing for a single screen (or just for screens, since we include different interaction modalities such as speech interaction and air gestures), it was very difficult to assess the User Experience (UX) of different parts of the room, as we were unsure how second (or even third in some cases) screens should behave. For that purpose, a sophisticated tool was developed, which was named “The Wizard of AmI”. This tool immensely helped the design team toward (i) creating interactive prototypes for an intelligent space, (ii) interacting with the generated prototypes both in the real and the simulated environment, (iii) assessing the selected interaction modalities and the behavior of various artefacts during a specific task, and (iv) exploring ideas for parts or artefacts of the environment that do not exist yet.

Following an iterative design process, before proceeding with the implementation, user experience experts and end users evaluated the developed prototypes (with the help of “The Wizard of AmI”), providing valuable feedback and identifying usability and UX-related issues early in the design process. The revised prototypes were subsequently implemented by the development team. As soon as a subset of the envisioned functionality was ready (i.e., ambient applications, intelligent artefacts and services), the operation of every component and the overall environment’s behavior was validated via AmITest [50]. Finally, a series of user-based evaluation experiments were conducted in order to draw insights by observing the users interacting with the living room environment (Section 5).

Designing and developing applications for the “Intelligent Living Room” is an ongoing process, since many of the available technologies are not merely newer, or faster, or better versions of established paradigms, but rather entirely new paradigms of interaction that are predicted to dominate the mainstream in the coming years (e.g., augmented/virtual/mixed reality applications). To this end, the hardware setup that currently empowers the “Intelligent Living Room” (Section 4.1) was defined in part according to the needs of the ambient applications developed to support the envisioned scenarios (Section 3.2 and Section 4.3), but also influenced by past or ongoing projects of the Ambient Intelligence Programme (http://ami.ics.forth.gr/) of the Institute of Computer Science of the Foundation for Research and Technology – Hellas (FORTH-ICS). However, our approach encourages a continuous strive to integrate emerging technologies, which not only improve performance and/or accuracy, but also introduce new opportunities to the already complex mix of fluid factors that influence design and development.

### 3.2. Motivating Scenarios

Scenario building is a widely used requirements elicitation method [112] that can systematically contribute to the process of developing requirements. In the case of the conceptualization and design of the “Intelligent Living Room”, it was decided that scenarios would offer an opportunity to empathize with potential users (i.e., personas) and express ideas in specific contexts. Having prior knowledge—through the development of past applications—of the capabilities of modern interaction modalities, applications and contexts (such as surface computing, second screens, or user tracking, which are relevant to the scenarios presented here) allowed team members to envision particular tasks and usage patterns that were incorporated into the scenarios. These scenarios were later distributed to the designers and developers as a tool to help them understand the context of use and envision what the interaction with various applications might look like and what modalities would be natural to employ. Finally, the scenarios became a reference to guide discussion and fuel brainstorming sessions. It is worth noting that during the writing of the scenarios, it became immediately apparent that even though the focus was on a particular part of the home (i.e., the living room) there were many home services that would have to be room-independent (Section 4.2.4). The following sections present three of the envisioned scenarios.

#### 3.2.1. Three-Member Family

On Monday afternoon, the father of the family, John, is returning home after a business trip, while his wife Anne is at work and their daughter is attending a French class. After a busy day, John decides to relax in the living room in front of the TV; he uses the universal remote control to turn on the coffee machine and prepare coffee. While he browses through the latest news of the day, the system—knowing his busy schedule—starts playing his favorite music to create a relaxing atmosphere. After a while, he decides to resume watching the movie that he left unfinished last night. Finding the movie is easy, since the system displays at the top the recently viewed movies and series. John uses the sensors embedded on the sofa to start the movie and increase the volume. The system dims the living room lights to create an appropriate setup and John starts enjoying the movie. After a while, the doorbell rings; the movie is paused and a live feed from the entrance appears immediately on the coffee table in front of him. It is John’s teenage daughter Helen, coming home from her French lesson. John stands up and moves toward the entrance; at this point, the system identifies his movement and increases the intensity of the room lights. John opens the door, greets Helen, and asks her how her day was. Helen seems stressed, she explains to her father that she feels disappointed because she performed poorly at her course exam. John tries to calm her down and moves to the kitchen to make her a cup of tea, while Helen sits on the sofa to catch her breath. In the meantime, her smart watch has been monitoring her stress level and given that the measured values were way above the threshold for quite some time, it initiates her personal relaxation program in the “intelligent” living room. The TV presents a calming video of a beach, the speakers play ocean sounds, the living room light intensity decreases and takes a blue hue, while the wall projector turns on to display similar images on the wall above the TV.

#### 3.2.2. One Home Resident and Two Guests

Chris, after returning home from work, enters the living room to relax in front of the TV. The intelligent environment detects his presence and automatically turns on the TV and the technologically enhanced coffee table. Chris moves his left hand over the sensor installed in the left arm of his sofa and performs mid-air gestures to navigate to the latest news of the day. At this point, the intelligent living room dims the lights and changes their color to a warm yellow hue in order to facilitate reading. While he browses the local news, he stumbles upon a breath-taking reportage regarding a robbery at a store in his neighborhood, and selects it to read more. Then, the TV starts playing the relevant video footage, while the coffee table in front of him presents more information about the incident, such as exact location, time, testimonies from local eyewitnesses, etc. 

After a while, the doorbell rings, and Chris get notified via a popup projected on the coffee table that his friends Jill and Ada came to pay him a visit. He then moves his hand over the controls included in the popup, and touches the “Open Door” button. As soon as Jill and Ada enter the room, they greet Chris and decide to watch a movie. Jill interacts with the TV (through the sensors installed in the sofa) in order to browse through the available movies. A rich movie library is displayed, and each time she selects a movie, the coffee table and the wall above the TV get filled with complementary information about the scenario, casting, ratings, awards, etc. The three friends decide to watch the movie *Inception*, so Ada leans forward and touches the play button, which is projected on the coffee table. The “intelligent” living room dims the lights completely, and the movie starts. At this point, the table and the wall lower their luminosity but continue to display live information regarding important scenes of the movie (e.g., actors taking place in the scene, location of the scene, trivia, soundtrack information, etc.). While watching the movie, Chris decides to offer a beverage to his friends, so he gets up and moves toward the kitchen. Then, the movie is paused automatically, and the lights increase their brightness. After a while, Chris returns to the living room holding three glasses of soda; he places them on the coffee table and sits on the sofa. Soon after the movie is resumed, the interface of the coffee table gets rearranged in order to display the available information in areas that do not get hidden by the glasses.

#### 3.2.3. Family with Young Children

The family gathers around the sofa to watch a family movie. Mom and dad sit on the sofa, while their two children, Sam and Sophie (10 and 8 years old, respectively) sit on the left and right side of the coffee table on their favorite bean bag sacks. 

Using the tablet, dad turns on the TV. The system is aware that the entire family is gathered around the coffee table, so the recommendation list of movies and series is automatically filtered to display family-suited material only. The kids as usual do not agree on which movie to watch, so mom suggests they play a game of ping pong on the coffee table in order to determine who gets to choose the movie. The kids agree, so mom says “HOME START PING PONG”, and immediately the game appears on the table. The information that was previously displayed on the coffee table now migrates on the wall above the TV. 

Sophie is concentrated on the table surface, and as soon as the game starts, she uses her glass of soda to throw the digital ping pong ball toward Sam, catching him by surprise and scoring the golden point that allows her to win the game and pick the movie. “Home PLAY *RATATOUILLE*!” she says happily, and the system dims the lights and begins the movie. 

While the movie is about a quarter of the way through, a subtle notification appears on the coffee table near where dad is sitting. Taking a look at the message, he realizes he will have to go upstairs in his office to have a private video call with a co-worker in order to help with a problem that has suddenly come up. 

Mom grabs the opportunity to take a look at the meals that the Home suggests for the day. She selects the “snack and food” option at the coffee table menu and browses recommendations based on the family’s preferences, including take-away options that the family has given a high mark in the past. Feeling too tired and too cozy to make the suggested bacon and lettuce sandwiches, she decides to pick a specific take-away option. The system automatically sends the order to the online store and sends a private code along with the order that the deliveryman can use to access the outside gate of the house. Payment is automatically handled, since all payment details have been verified as legitimate the first time the family ordered from that restaurant. An additional 10% tip is included after a prompt by the system.

Dad returns from the business call and sits back on the sofa. The coffee table displays the movie player’s progress bar, since dad has already pressed START on the remote control. Twenty minutes later, the delivery guy has arrived. He has used the temporary code—like he always does—to open the outside gate. The movie pauses automatically, and a live feed from the entrance is displayed on the coffee table. Dad gets up to open the door and grab the food, returning and placing all the containers on the coffee table. The information that was displayed on the coffee table is now re-arranged (both on the coffee table and the wall above the TV) to make space for the food.

### 3.3. High-Level User Requirements of the Intelligent Living Room

This section presents the high-level requirements that an “Intelligent Living Room” should satisfy, which are categorized under two categories: namely, Functional and Interaction requirements. The requirements have been collected through an extensive literature review and an iterative elicitation process based on multiple collection methods such as brainstorming, focus groups, observation, and scenario building, as described in Section 3.1. Note that some of them are specializations of inherited high-level requirements originating from the overall Smart Home environment, which are propagated at a room—sub-environment—level (e.g., user identification and tracking, interaction modalities common throughout the smart home).

**Functional Requirements**

REQ1. Identification of users in need in order to provide appropriate assistance help
REQ2. Delivery of various useful information (e.g., weather, calendar, traffic, etc.)
REQ3. Delivery of information gathered from various home services (e.g., room temperature, estimated time of dinner preparation, current energy consumption level)
REQ4. Hosting of applications that facilitate entertainment, communication, and socialization (e.g., Movie player, Newsfeed player, Photo gallery, Video call)
REQ5. Hosting of various document viewer applications
REQ6. Support of single or multiplayer games
REQ7. Support of collaborative activities with remote users (e.g., playing a game or watching a movie with a friend)
REQ8. Presentation of secondary information depending on the current activity (e.g., display actors and reviews while watching a movie)
REQ9. Notification delivery through the most appropriate medium depending on the privacy settings and their priority
REQ10. Automatic control of ambient room facilities (i.e., blinds, lights, A/C) depending on the current conditions
REQ11. Automatic adaptation of the room atmosphere (i.e., lights, music, aroma) depending on the context of use (e.g., while watching a movie or while relaxing)
REQ12. Provision of appropriate recommendations depending on the current activity (e.g., suggest a program while watching TV)
REQ13. Connection with popular social media (e.g., suggest current movie to a friend)
REQ14. Support of online shopping (e.g., food delivery, order a book, rent a movie)
REQ15. Remote control and monitoring of all home facilities (e.g., devices, appliances, surveillance system)
REQ16. Assurance of efficient energy use (e.g., turn off TV when everyone leaves the room)
REQ17. Provision of appropriate user-friendly tools for programming the ambient facilities of the house
REQ18. Synchronization of user activities amongst rooms (e.g., when the user moves from the living room to the bedroom—for a significant amount of time—the music plays at the appropriate speakers, as if it follows his path)
REQ19. Provision of inhabitant’s safety (e.g., children monitoring to prevent hazardous situations such as “touching a power outlet”)
REQ20. Assurance of respecting user preferences
REQ21. Automatic learning and adapting to user habits
REQ22. Assurance of protecting personal information from house guests
REQ23. Ability to deactivate the ambient facilities of the room on demand

**Interaction Requirements**

REQ24. Support natural language voice input (e.g., through situated embodied virtual agents)
REQ25. Support natural hand and body gestures (e.g., wave goodbye to turn off the lights and put all the devices to sleep)
REQ26. Support touch interaction (e.g., touch on top of surfaces that double as screens, such as a coffee table)
REQ27. Support interaction through personal devices (e.g., smartphone, smart watch)
REQ28. Use the walls as large displays
REQ29. Transform everyday objects (e.g., coffee table, sofa) into interactive surfaces
REQ30. The User Interface (e.g., look and feel, theme, location) and the interaction paradigm (e.g., lock the door with a vocal command) should be used consistently around the house
REQ31. Design the sitting area appropriately to accommodate family and guests
REQ32. Design the sitting area appropriately to support collaboration and games
REQ33. Support of fluid furniture layouts suitable for heterogeneous activities (e.g., a board game with friends, watching a movie, cleaning the house)

## 4. The Intelligent Living Room

Ambient Intelligence (AmI) environments [113] are expected to transparently interact with the users either passively, by observing and trying to interpret their actions and intentions, or actively by learning users’ preferences and adapting their behavior accordingly to improve the quality of life. Particularly, according to [114], “AmI is a user-centric paradigm, it supports a variety of artificial intelligence methods and works pervasively, non-intrusively, and transparently to aid the user”. In order to be able to act as described, the architecture of an AmI environment should consist of four main layers [115], namely: (i) Sensing, (ii) Networking, (iii) Perception and Reasoning, and (iv) Acting. It is obvious that such environments evolve around the needs of their users, and their main objective is to act in an appropriate manner when deemed necessary. Indeed, in many application domains such as Ambient Assisted Living (AAL), eHealth, Domestic Life, Learning and Education, it is imperative to provide the right type of help or support as soon as the user needs it.

The “Intelligent Living Room” (Figure 1b) is part of the “Intelligent Home” in vitro simulation space located at the AmI Facility within the FORTH-ICS campus. Inside intelligent environments, everyday user activities are enhanced with the use of pervasive and mobile computing, sensor networks, artificial intelligence, multimedia computing, middleware, and agent-based software [116]. The following sections describe (i) the hardware facilities of the living room environment, which include both commercial equipment and technologically augmented custom-made objects, (ii) the software infrastructure, which includes sophisticated middleware, monitoring, and decision-making mechanisms and distributed microservices that compose the Ambient Intelligence core, (iii) the applications developed specifically for the “Intelligent Living Room”, and (iv) the high-level principles that govern the applied interaction paradigm(s).

### 4.1. Artefacts of the Intelligent Living Room

In the context of Ambient Intelligence, equipping the physical environment with technologically enhanced artefacts is a challenging task. In practice, such artefacts should be unobtrusive, hidden, or embedded in traditional surroundings and furniture so that they minimize their overall footprint and fit into the environment from an aesthetics point of view. Adhering to these principles, the “Intelligent Living Room” introduces a collection of interdependent artefacts that have the ability to communicate, creating a unified interaction experience. These artefacts fall under two categories:

1. Common domestic equipment, such as a wide variety of commercial devices (e.g., Philips Hue Lights [117], smart locks [118], blinds [119], Alexa [120], oil diffuser [121], a wearable device that offers real-time physiological data acquisition [122]) and appliances (Humidity, Ventilation, Air-Condition—HVAC) that can be controlled either via their own Application Programming Interface (API) or using dedicated solutions (e.g., KNX bridge [123]).
2. Technologically augmented everyday objects, such as AmITV, AugmenTable, SurroundWall, and SmartSofa. The characteristics of these intelligent artefacts are described in the following Section 4.1.1, Section 4.1.2, Section 4.1.3 and Section 4.1.4.

#### 4.1.1. SmartSofa

**Figure 2:**
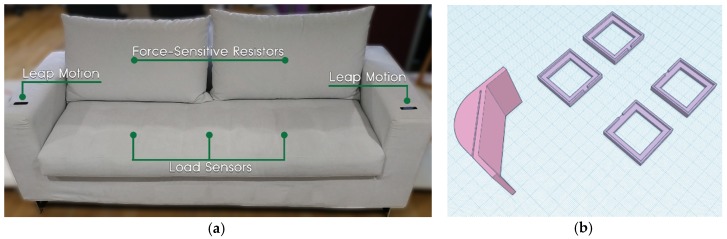
(**a**) SmartSofa artefact; (**b**) 3D printed cases for installing the sensors into the sofa.

SmartSofa (Figure 2) is a commercial sofa equipped with various sensors. A number of force-sensitive resistors and load sensors (all connected to an Arduino microcontroller [124]) are installed in the sofa’s back and under its bottom pillows, respectively. The purpose of this installation is two-fold: on the one hand, it assists the detection of user presence inside the room, and on the other hand, it provides information regarding the user’s posture (i.e., the user leans back or forth) and position (i.e., middle, left, right) while seated. Moreover, two Leap Motion sensors [125] are embedded in each of the sofa’s side arms, providing an invisible input control that enables users to easily manipulate the overall interactive environment through mid-air hand gestures.

#### 4.1.2. AmITV

The AmITV [126] artefact comprises a 70-inch 4K smart TV and a software shell that can host and manipulate interactive applications. TVs are usually positioned in the middle of the living room facing users directly at their eyesight level, have higher resolution, and deliver more accurate colors and sharper picture compared to a projector. Therefore, AmITV constitutes the main display of the “Intelligent Living Room”, while AugmenTable and SurroundWall act as secondary screens, as we will see in the respective sections. 

The interactive applications on AmITV (e.g., Movies, Slideshow, News) mostly aim to entertain and inform the user; nevertheless, applications of general interest are also available, such as the Home Controller [127], the AmI Garden Controller [128], etc. 

From an interaction perspective, AmITV features special software that enables the incorporation of multiple input and output channels, thus permitting interaction even when a user’s primary channel is occupied, unavailable or non-existent. Indicatively, a Kinect sensor [129]—located on the top of the TV—and motion sensors mounted on the ceiling overlook the entire area of the living room, thus enabling AmITV to monitor the presence of people, track their movements in the surrounding environment, and adjust the interaction paradigm accordingly (e.g., display controls on the table’s surface when the user leans forward) and the functionality (e.g., lock/unlock the TV, pause the movie player, hide private messages). Additionally, the Leap Motion sensors embedded in both arms of the SmartSofa artefact permit users to control the applications running on the AmITV artefact by simply moving their hands and fingers, without having to reach for the remote. 

#### 4.1.3. AugmenTable

**Figure 3:**
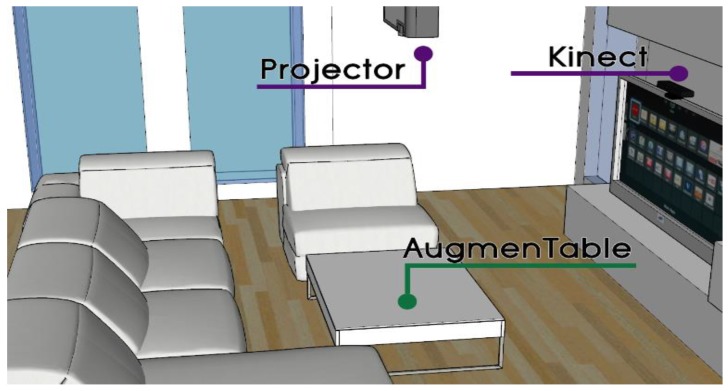
AugmenTable consists of a projector embedded on the ceiling above the coffee table, and a Kinect sensor installed on top of the TV.

AugmenTable (Figure 3) is a stylish commercial 52 × 35 inch coffee table made of wood with a smooth, non-reflective white finish, which in addition to its intended use for placing objects on top of it (e.g., cups, plates, books), acts as a large projection area where secondary information can be presented from a small form factor projector embedded in the ceiling. Its physical attributes (i.e., color, dimensions) and placement (i.e., in front of the couch) enable users to clearly see the projected content on demand without being overwhelmed when interacting with the main display (i.e., AmITV).

Through a second Kinect sensor installed on top of the TV facing directly at the table’s surface, AugmenTable becomes a touch-enabled surface that can recognize the physical objects placed on it as well. Additionally, a vibration motor is located under the table in order to provide haptic feedback to the users when deemed appropriate, e.g., deliver a silent alarm that indicates food delivery is on its way (e.g., three short vibrations) when the TV is playing a critical scene and the notification service decides not to explicitly interrupt the user. Finally, the force-sensitive resistors and load sensors embedded in SmartSofa’s seat and back permit the monitoring of the seated users as well as their posture while seated. Depending on whether the user leans toward the table, relaxes on their back, or lies down completely, the information displayed on AugmenTable, as well as the available interactive controls, adapt their appearance and/or their location to better accommodate the user’s needs (e.g., interactive controls remain hidden when out of reach, the brightness level is set to low when the user is not looking toward the AugmenTable).

#### 4.1.4. SurroundWall

The SurroundWall artefact comprises a short-throw projector embedded in the ceiling above the TV. This installation transforms the wall around the TV into a secondary non-interactive display, providing an enhanced viewing experience by augmenting—in a context-sensitive manner—the content presented on the AmITV artefact. For example, when the user is watching a soccer match on the TV, the wall couples the viewing activity with second screen interaction [130] by presenting additional information such as game summary, team rosters, advanced players’ stats, live score from other matches, relevant social media updates (e.g., tweets), etc. Finally, SurroundWall, along with other ambient facilities of the “Intelligent Living Room” (e.g., speakers, aroma diffuser), can deliver immersive experiences—which the user will perceive as being physically present in a non-physical world—by projecting real world environments to the surrounding wall. Since exposure to a natural environment is considered as one of the most effective techniques for relaxing a user with high stress levels [131], this type of functionality offered by SurroundWall can be utilized for creating a relaxing atmosphere—when necessary—based on the current user needs and context of use. 

### 4.2. Software Infrastructure

#### 4.2.1. Ami-Solertis

Building services for Ambient Intelligence environments implies that multiple different technologies and protocols will be used by the various technological components in order to define and expose their functionality. The deciding factor on which specific protocol will be used, in addition to any prospective standards and guidelines that suggest certain approaches [132,133], is their technical capabilities from a hardware (e.g., network interfaces, processing power, battery-based operation) and a software (e.g., OS, runtime environment) perspective. 

AmI-Solertis [134] enables the fast, easy and error-free integration of external AmI artefacts (i.e., services) independently of their type (i.e., back-end, front-end, or mixed services that follow the Software-as-a-Service paradigm [135]). Moreover, it supports the creation of AmI scripts that define the behavior of the technological facilities (i.e., business logic) toward creating pervasive, intelligent, and personalized environment experiences by combining multiple components. The AmI-Solertis system is built using a microservice architecture style that enables it to be used as a backbone [136] across a wide range of ubiquitous systems [137] and intelligent environments with diverse objectives (e.g., compose a new compound service using existing ones, define the behavior of a smart hotel room [49], control a smart home, build an intelligent management system for a smart city [138]). 

**Figure 4:**
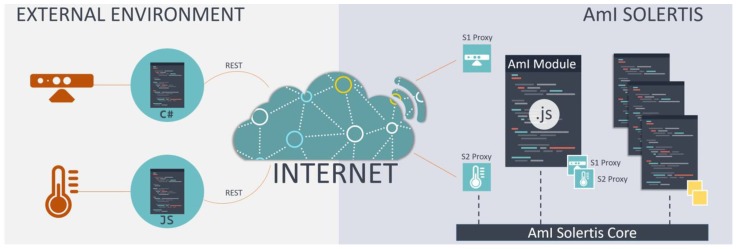
The AmI-Solertis Hybrid Communication protocol.

Building on the benefits of asynchronous and event-based communication [139,140,141], AmI-Solertis introduces a unified Hybrid Communication protocol that combines the widely used Representational State Transfer (REST) [142] and the OpenAPI Specification (OAS) [143] with asynchronous and event-based communication facilities to integrate heterogenous services in a standardized—yet agnostic—manner (Figure 4). Therefore, an AmI artefact or an AmI script on the one hand exposes a REST interface to receive incoming calls, and on the other hand communicates its intention to the AmI ecosystem by emitting appropriate events via the AmI-Solertis Event Federator.

AmI-Solertis encapsulates the complexity of configuring and performing remote calls through automatically generated proxies that eliminate the difficulties of distributed programming by (i) masking remote operations into local methods and (ii) enabling consumers to register their interest to events coming from a remote component without specifying any details about the underlying topology and infrastructure. In addition to code minimization, proxies also empower AmI-Solertis to dynamically adapt and adjust the invocation process in order to address emerging requirements such as re-routing a call to a replicated host to achieve load balancing, immediately terminating a call if the remote endpoint is unavailable, intercepting a call and logging relevant Quality of Service (QoS)-related metrics, and replacing a target endpoint with another that offers semantically similar functionality. In addition to AmI components management (i.e., AmI artefacts or scripts), AmI-Solertis offers an online Integrated Development Environment (IDE), named AmI-Solertis Studio, which aims to assist developers in creating, exploring, deploying, and optimizing the AmI scripts (i.e., programs) that control the behavior of the AmI environment by combining and orchestrating various AmI artefacts or other AmI scripts that reside in the ecosystem.

#### 4.2.2. LECTOR, LECTORstudio, and ParlAmI

LECTOR [144] is a framework that takes advantage of the ambient facilities already existing in AmI environments in order to identify when the users need help or support and intervene to improve their quality of life. It follows the trigger-action model [48,145,146], which has been in the spotlight as a form of programming AmI environments, using simple “if then” rules. LECTOR introduces a three (3) step process for connecting behaviors with interventions. The first step is to define a behavior, the next step is to describe the conditions under which the behavior becomes a trigger, and the last step is to connect it with an intervention.

Even if this decomposition increases the number of steps that a user must complete in order to connect a trigger to an intervention, it offers scalability and better rule management. In particular, the three necessary elements (i.e., behavior, trigger, and intervention) are defined in isolation and are only connected in terms of their outcome. Therefore, any element can be modified independently of the others, and as long as its outcomes remain the same, no more adjustments will be required for the system to continue to operate as prior to the change. This approach not only minimizes unwanted ramifications, but also facilitates collaboration as new rules can be easily created by different users, given that their “connection points” will always be their outcomes. This is inspired by how an Application Programming Interface (API) simplifies programming and enables computer programs to evolve independently by abstracting the underlying implementation and only exposing objects the developer needs. The core concepts of this rule-based approach are explained below:

- Rule is a model that binds a behavior with an intervention via a trigger.
- Behavior is a model that represents the actions of a user or a device (e.g., a user yells or sleeps).
- Trigger is the model of a high-level behavior that can initiate an intervention.
- Interventions are system-guided actions that aim to help or support users during their daily activities.
- Intervention hosts are the artefacts of the environment that can (i) either display an application (with carefully curated content) or (ii) control the physical environment.

Developers and non-technical users can easily and rapidly create such rules through a sophisticated user-friendly authoring tool named LECTORstudio [147]. In more detail, LECTORStudio’s intuitive UI enables developers to integrate the building blocks necessary for programming the intelligent living room, while it also permits the house residents to create their own scenarios and customize LECTOR’s decision-making process according to their needs. 

As an alternative to LECTORstudio, which uses a Graphical User Interface (GUI) for creating the necessary “if–then” rules to program an AmI environment, the potential of conversational interfaces (CIs) was also investigated, resulting in the creation of ParlAmI [148], a multimodal conversational interface. ParlAmI introduces a hybrid approach that combines natural language understanding (NLU) with semantic reasoning and service-oriented engineering so as to deliver a multimodal CI that assists its users in defining the behavior of intelligent environments. Particularly, it offers an alternative easy-to-use approach toward generating such rules (especially for novice users with little or no programming experience) through conversing in natural language with a context-aware intelligent virtual agent (i.e., chatbot).

#### 4.2.3. UInify

UInify is a framework that aids designers of intelligent spaces in delivering unified experiences to the end users. Namely, it offers (i) a collection of tools that enable the visual combination of several individual UIs toward introducing new User Interface compositions and (ii) a universal style guide guaranteeing that the generated UIs share a common and consistent look and feel across all devices. UInify embraces the key concepts of User Interface Mashup web technologies [149] and User Interface composition [150] in order to introduce new unified applications that incorporate several UI components into a common presentation layer for all underlying devices and services that can interconnect in the background. Additionally, UInify’s ability to control multiple connected devices simultaneously enables the integration of several input and output devices, thus achieving a seamless multimodal user experience. Overall, UInify allows the Intelligent Home to have several distinct software components developed independently by different experts, with the presentation layer being orchestrated under a common roof. This approach offers several benefits such as:

- Maximum reusability: Developers do not need to build a single unified application from scratch; instead, they can compose the desired UI by using existing functionality offered via separate web applications.
- Reduced development cost: By re-purposing existing software, developers can create composition at a fraction of the time they would require to re-implement everything.
- Enhanced configurability: UInify enables the creation of an unlimited number of compositions, which get activated in a context-sensitive manner. Therefore, the same components can be used differently toward addressing different problems; for example, when presented on the SurroundWall, the Slideshow and the Chat Application can display the albums and contacts menu respectively, whereas when viewed on the AugmenTable while having dinner, the composition’s footprint would get much smaller by keeping only the essential information (e.g., current chat and image).
- End-user customization: End users can create their own custom compositions, using any of those available, to match their needs and preferences.

#### 4.2.4. AmIHomeOS

AmIHomeOS is the core framework behind the “Intelligent Living Room” that aims to transform the space into an all-inclusive environment that assists users in an “intelligent”, personalized manner. To that end, it is built having in mind a slightly adapted version of the principles that govern a modern operating system [151]; that is, to provide AmI Scripts (i.e., programs that define the behavior) with a better, simpler, cleaner, model of the environment and to handle managing all the hardware (e.g., input devices) and software (e.g., diet preferences) resources. Moreover, AmIHomeOS permits every ambient application to define its own AmI Scripts that describe how its functionality is going to be adapted according to the current context and user preferences (e.g., user’s mood, presence of others, task at hand).

In more detail, AmIHomeOS (i) enhances interaction by applying multiple modalities, (ii) exploits contextual information to make “informed” decisions, (iii) personalizes the delivered content according to users’ preferences, and (iv) exposes the functionality of its intelligent artefacts as a service in order to enable integration with third-party tools. From an engineering perspective, AmIHomeOS follows the principles of a microservice infrastructure [136,152], similarly to most of the latest applications/frameworks targeting intelligent environments [153]. That way, it (i) ensures interoperability and loose coupling between the various heterogeneous and distributed services/components by endorsing the Software-as-a-Service (SaaS) model [135], and (ii) enhances scalability, offers faster development, and lowers the cost of changes/updates [152]. 

AmIHomeOS can be seen as a collection of distributed, isolated microservices that operate autonomously, but expose their functionality—of common interest—over REST (according to AmI-Solertis principles) to be used as part of the wider intelligent ecosystem of AmIHomeOS. A comprehensive list of the currently available services can be found below:

- User preferences and profile: provide access to the list of personal data, characteristics, and configuration parameters for every user (e.g., resident, domestics worker). User models are expandable structures, so as to enable ambient applications to augment them on demand. This information can be exploited by the other services and/or ambient applications in order to consider the persons’ characteristics and preferences.
- Occupants tracking: monitors the presence of people and tracks their movements (and actions) in the surrounding environment.
- User activity tracking: keeps track of the activities that a user is engaged with; for every activity, the completed, ongoing, and future steps are available (e.g., step 7 out of 15 in preparing dinner).
- User health state and mood tracking: by monitoring various sensors (e.g., wearables) and user activities, this service stores—in a timely manner—various health-related information (e.g., amount of physical activity, stress level) and exposes both aggregated and detailed (recent) data.
- Home Context Manager: holds current “public” context (i.e., state) of every application/device/service that is part of the intelligent environment (e.g., bed-side lamps are on, cake should be baked for seven more minutes).
- Calendar/Agenda: allows an ambient application to access and modify the user’s appointment, tasks, meetings, and events.
- Food- and diet-related services: these services are part of the “Intelligent Kitchen” parallel project and are mostly used to accommodate cooking activities (e.g., nutrition facts, ingredients availability, recipes inventory); nevertheless, in the context of the “Intelligent Living Room”, they provide information to any interested application (e.g., CaLmi) or AmI Script (e.g., “Do not forget your lunchbox before you leave for work”).
- Local weather conditions: provide access to weather data that can be used for clothes recommendation, personalized notifications (e.g., “carry an umbrella today”, “do not forget to apply sun screen”), etc.
- Screen-time tracking: aggregates the amount of time spent interacting with the various ambient applications of the “Intelligent Living Room” in order to be used by activity recommendation systems (e.g., fitness advices, tips to improve “sleep hygiene”).

### 4.3. Ambient Applications and Services

#### 4.3.1. Entertainment Applications

**Figure 5:**
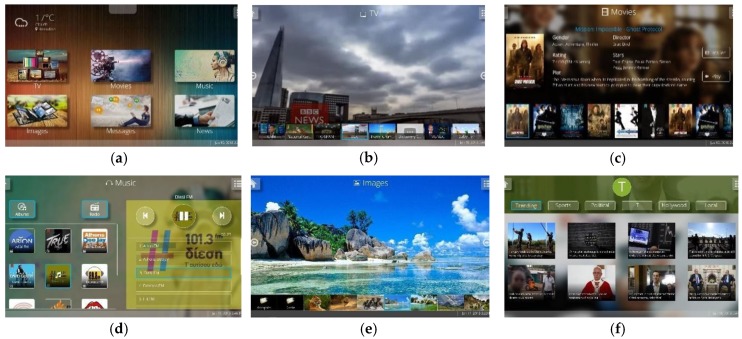
AmI TV instances: (**a**) Home Screen; (**b**) TV application; (**c**) Movies application; (**d**) Music application; (**e**) Images application; (**f**) News application.

A set of entertainment applications—similar to those offered by trending Smart TVs or media centers—were created for the “Intelligent Living Room”. These applications (Figure 5) employ various contextual information (e.g., users’ preferences, habits, daily routine, location in space) to make informed decisions and intelligent recommendations to their users. Specifically, (i) a TV application hosts a plethora of TV channels that provide live-streaming via either their YouTube channels or their official sites, (ii) a Movies application permits users to browse through and watch movies and TV series from the home’s or a remote cloud server, (iii) a Music application contains both personal music albums as well as radio stations that support live-streaming, (iv) a Slideshow application allows users to view their personal photos, permits categorization into custom folders, and supports the creation of shared folders that can be accessed by all family members, (v) a News application enables users to browse through collections of news feeds from all around the world, and (iv) a Games application hosts games available online [154].

All applications can be launched either on the main display (i.e., AmITV) or on the secondary ones (i.e., AugmenTable and SurroundWall). However, apart from their main components (e.g., video player, image viewer), these applications also consist of various components that host secondary information. The latter can be distributed to AugmenTable and SurroundWall, depending on the context of use. For example, the movie application incorporates a “home theatre” component that enables users to browse through the available movies, see details regarding the actors, etc.; when the user is watching a movie on the AmITV artefact, AugmenTable displays live information regarding the current scene.

Additionally, every application automatically filters its multimedia content to match the interests, likes, dislikes, and preferences of the current user, whereas when children are present, the system hides inappropriate content (e.g., movies, news). To that end, an internal tag-based classification scheme personalizes the recommendation/filtering process [155,156]. Each user can add channels, movies, albums, images, etc. to their favorites, which are displayed in a prominent place for fast access, while when two or more users decide to interact as a group (e.g., watch a movie, listen to music), the system can make suggestions based on their common interests. Finally, when applicable (i.e., movies, songs), appropriate indications inform the users about items that they have paused viewing or listening, while dedicated controls permit immediate resuming.

These applications are also accompanied by a set of behavior scripts that improve UX. Indicative examples are:

- Automatic control of physical and ambient lighting
- The music stops as soon as a user starts watching TV
- The movie pauses when the user leaves the living room area
- Personal content becomes available or remains hidden based on each user’s preferences when multiple users are simultaneously present
- When the user is away from the room, the system reads out loud any notifications

#### 4.3.2. Notifications

The “Intelligent Living Room” features a sophisticated notification mechanism that exploits various contextual information (e.g., user’s profile, user’s agenda, user’s location in the house, presence of multiple users, current activity) in order to provide notifications (e.g., medicine reminders, cooking alarms, work-related updates, burglar alarms) in a timely and space-aware manner. Furthermore, the system takes into consideration each user’s privacy settings to decide when, where, and how a notification will be displayed, particularly when other users are also present.

Specifically, there are three types of notifications categorized by their priority: (i) low-priority notifications of little importance or urgency (e.g., movie download complete, laundry is done), (ii) medium-priority notifications that must capture the user’s attention (e.g., incoming message from a colleague), and (iii) high-priority notifications that must pause the current activity of the user so as to gain his focus (e.g., cooking is done). 

These notification types can be presented either as toast or popup messages on each of the available living-room displays (i.e., AmITV, AugmenTable, and SurroundWall). The term toast notification describes small messages that show up in a box at a specific location of the display and disappear automatically after few seconds. It is usually employed to inform users about events that do not require specific attention/action. Toast notifications for low and medium-priority messages are presented visually through any available display. The box emerges on a subtle area (e.g., on the top-right for the AmITV), while the duration that it remains visible varies depending on the context of use (e.g., user activity, type of message). In case of medium-priority messages, short sound effects are also used to capture the user’s attention.

**Figure 6:**
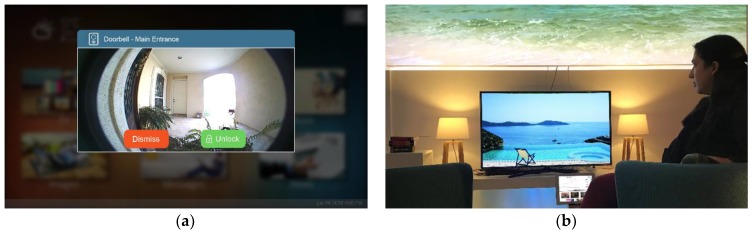
(**a**) Instance of a popup notification displayed on the AmITV artefact; (**b**) The environment assists in reducing the stress of the user (CaLmi).

On the other hand, popup notifications (Figure 6a) are used to deliver high-priority messages. A small window appears in the middle of the display on the foreground of the visual interface, thus ensuring that the user will get the message; for example, if the user is watching a movie in the living room and the baby wakes up and starts crying in the bedroom, AmITV automatically pauses the movie and displays the live feed from the nursery. Contrary to a toast notification, a popup window does not disappear automatically, but requires the user to perform one of the available actions (e.g., OK, Unlock door, Turn off the oven) or dismiss it. Apart from text messages, the popup window can also display rich user interfaces such as live-streaming video from the intelligent environment, videos, and images, etc., thus improving the overall UX.

#### 4.3.3. Communication Application

A multimodal family communication application was created, allowing users to communicate with each other, both in real-time and asynchronously. In more detail, users can communicate with other individuals or with multi-member group channels, either via text or multimedia messages, following a multimodal approach. Each family member can receive the messages intended for them on any of the available living-room displays (i.e., AmITV, AugmenTable, and SurroundWall). In order to send a text message, they can use the hardware keyboard embedded in the remote control or the software keyboard of their personal smartphone. Additionally, through the application UI, the users have access to “quick responses” that are either created by them a priori or suggested by the system based on frequently sent messages, hence facilitating one-click interaction. 

Apart from text messages, the application enables users to communicate through images, video, and voice recordings, thus enriching textual information with visual and vocal content. Such media can be recorded either via a smartphone or the Kinect device mounted on top of the TV. 

Finally, the family communication application, amongst others, introduces a behavior script that improves UX in terms of privacy. In particular, knowing where everyone is sitting in front of the TV, the system can identify who requested to read his/her messages and presents them—skipping any unnecessary authentication steps—if the relevant privacy settings permit such an action (e.g., other co-located users have the right to view them).

#### 4.3.4. Home Control

The “Intelligent Living Room” features a mechanism that permits it to host various interfaces for controlling the facilities of intelligent environments in any of the available living-room displays (i.e., AmITV, AugmenTable, and SurroundWall). Each artefact can present adapted versions of the available home control interfaces according to its characteristics (e.g., input method, physical dimensions, resolution, brightness, distance from the user), through which users can manipulate the physical environment (e.g., lights, HVAC, blinds, oven). To ensure a consistent look and feel across devices, all applications follow the same set of design guidelines and interaction principles. In particular, all of them implement multiple variations that can fit in different settings (e.g., full screen, part of a UI composition, embedded in a popup), whilst a sophisticated UI engine collects, under a common roof, all the individual UIs and introduces new, rich, and context-sensitive UI compositions (AmIViews) in real time. For instance, if the doorbell rings (i.e., a high-priority event) while the user is watching a movie, then the movie will pause and the popup notification displays both the live feed from the front door and the appropriate controls to quickly unlock and open the door.

#### 4.3.5. CaLmi

CaLmi [157] is a pervasive system deployed in the “Intelligent Living Room” that aims to reduce the stress of the home inhabitants (Figure 6b). Particularly, the system aims to detect when a user is stressed and tries to help them relax by activating a relaxation program in the ambient environment. In order to do so, CaLmi is built on top of LECTOR (Section 4.2.2) and provides rules (i) that permit the identification of users who require support, and (ii) that define the interventions (i.e., relaxation programs) to be initiated depending on the situation. 

Identifying whether a user is stressed or not requires the combination of many parameters; in more detail, CaLmi uses the inhabitants’ wristbands so as to collect various physiological signals (i.e., electrodermal activity—EDA, heart rate—HR, interbeat interval—IBI, blood volume pressure—BVP, skin temperature—ST and accelerometer) that can potentially indicate high levels of stress. Additionally, various contextual data (agenda, household utilities bills, bank account balances) are utilized in order to better understand the user’s daily activities and disambiguate whether changes in physiological signals are caused by stressful events, unusual sleeping hours, physical activity, etc.

In order to provide ambient relaxation programs, CaLmi employs the applications described in Section 4.3.1, Section 4.3.2, Section 4.3.3, Section 4.3.4, Section 4.3.5 and Section 4.3.6. Particularly, whenever high stress levels are detected or the user just wants to relax, the system suggests an intervention with the most appropriate technique (e.g., diaphragmatic breathing, music therapy, visual exposure to natural environments) according to the current user needs and context of use. However, the process of selecting an appropriate intervention does not end by selecting a relaxation technique. The system must also decide on the appropriate hosts for the selected program. The available intervention hosts of the “Intelligent Living Room” that can be used for offering the relaxation techniques are the displays (i.e., AmITV, AugmenTable, and SurroundWall), the speakers, the lights, and the scent diffuser. After an intervention initiates, the users can optionally (de-)activate various features (e.g., lights, TV, wall projection).

The following brief scenario demonstrates how the system works. A gentle notification pops up on John’s smartphone informs him that his stress levels are high and asks if he wants to launch the “Exposure to Nature” relaxation program or choose one of his own. He decides to select the recommended one. Since he is alone in the living room, the system creates a relaxed atmosphere with a forest waterfall theme in that room. A video of a waterfall in the heart of the lush forest is displayed on the wall, and the light intensity decreases taking a blueish color, which resembles the color of the waterfall’s water; additionally, birds singing and sounds of running water are played from the room’s speakers, while the aroma diffuser fills the space with the scent of the Hinoki tree (Japanese Cypress). John can stop the program at any time, and upon completion, a notification popup will inform him about the new stress levels.

#### 4.3.6. Chatbot Application

As already mentioned, the software infrastructure of the “Intelligent Living Room” includes a conversational interface, which is named ParlAmI. ParlAmI does not rely only on text messages to receive user input; it also employs graphical user interface components so as to facilitate the communication process. A core set of custom conversation components were designed and developed based on visual components that users are already familiar with: (i) text, (ii) a group of buttons, (iii) text and buttons, (iv) an image with text and buttons, and (v) an image carousel with text and buttons. These UI elements simplify the user’s effort by limiting the required input to just a few options, as reducing the number of attention-grabbing elements simplifies the interface while strengthening the focus on what is actually important [158]. In order not to limit user freedom, ParlAmI also permits the users to provide their own input in case they are not satisfied with the suggested messages. 

**Figure 7:**
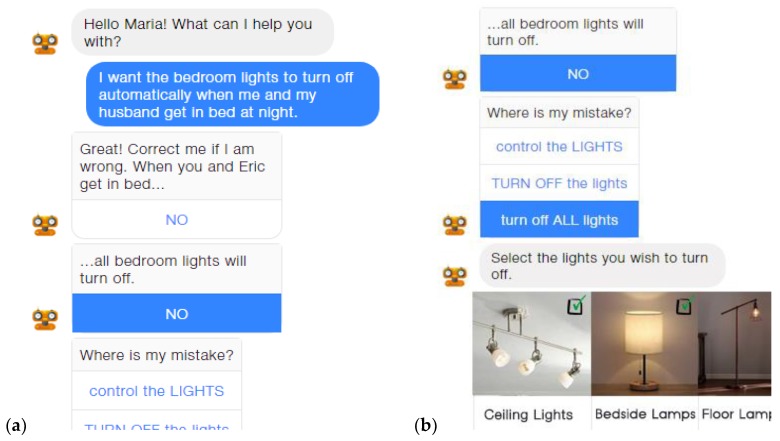
Chatbot application: (**a**) A message is decomposed into smaller chunks permitting the user to disapprove each one of them; (**b**) The user can correct a specific part of the rule(s), instead of having to repeat the complete conversation.

The conversational interface design for ParlAmI features various mechanisms trying to cope with misunderstanding on behalf of the system:

- Confirmation mechanism. Instead of asking the user continuously to (dis)approve every system action before asking the user a new question, the chatbot repeats the last acquired message, inviting the user to interrupt the conversation in case they identify a wrong assumption.
- Message decomposition. In case the user provides a complex message, the chatbot decomposes it into smaller meaningful chunks and then repeats them one by one, permitting the user to interrupt the conversation in case a misconception is identified (Figure 7a).
- Error recovery. A user disapproving a system statement means that the chatbot did not interpret correctly their intention. To this end, it initiates an exploratory process to identify what was the source of the problem and displays a “text and buttons” type of message asking the user what the mistake was (Figure 7b).

### 4.4. HCI Aspects

#### 4.4.1. Composition, Consistency, and Continuity

People are increasingly using an abundance of devices daily, and in many cases, these devices need to work together in a seamless manner toward achieving the same goal, which is known as cross-device interaction [159]. Regarding systems that have the ability to migrate amongst various devices, Rowland [160] wrote that it is not enough to design individual User Interfaces (UIs) for each device in isolation. On the contrary, the top priority should be to create a coherent understanding of the system, as well as a solid intercommunication between devices. In [161], three key concepts for cross-platform service User Experience (UX) are defined, which together ensure a coherent experience:

- Composition refers to the way the functionality of a service—especially the user-facing functionality—is distributed across devices. Good composition distributes functionality between devices to make the most of the capabilities of each device. Hence, designers have to figure out which device handles which functionality. Each device may have a different role in terms of providing user interactions, connectivity, information gathering, processing, or display. For example, AugmenTable is a great candidate to be used as an interactive second screen; however, its physical attributes (i.e., users sitting on the sofa have to stretch in order to reach it) make it less suitable for typing text.
- Consistency creates a sense of coherence of the overall system. It is important to make the devices look, feel, and sound like members of the same ecosystem, so that users form a clear mental model of the system and its capabilities. The ambient applications that have been developed for the “Intelligent Living Room” share a common style guide (i.e., UInify), which guarantees that the aesthetic and visual design is the same across all the devices. Additionally, during the design process, it was ensured that the interaction architecture (i.e., how functionality is organized) and the interaction logic (i.e., how tasks are structured or the types of control used) follow a consistent pattern.
- Continuity refers to the flow of data and interactions in a coherent sequence across devices. The user should feel as if they are interacting with the service through the devices, not with a bunch of separate devices. In order to achieve continuity, the AmIHomeOS infrastructure enables the ambient applications to be synchronized when they migrate among the various artefacts. Apart from data and content synchronization, in order to achieve continuity, cross-device interactions must be clearly signposted. Toward that direction, every ambient application or AmI Script that aims to support continuity follows the Pub/Sub model and relies on the Event Federator service (part of the AmI-Solertis framework) to monitor the various communication channels and act/react accordingly.

#### 4.4.2. Multimodality

One of the main characteristics of the “Intelligent Living Room” is the incorporation of multiple input/output channels that enable interaction even when a user’s primary channel is occupied, unavailable, or non-existent. In more detail, the following input modalities are provided:

- Virtual pointer. Users can control the TV interface by hovering their hand over the Leap Motion sensors, which are embedded in SmartSofa’s side arms. A virtual cursor that follows the movements of their hands enables them to focus on and select areas of interest.
- Mid-air gestures. Appropriate mid-air gestures, such as palm tilt, finger pinch, and hand swipe are also available in order to permit users to complete specific actions (e.g., volume up/down, next/previous item in a list, zoom in/out etc.) quickly and in a natural manner.
- Touch. Through the Kinect sensor installed on top of the TV facing directly at the coffee table’s surface, AugmenTable becomes a touch-enabled surface. Depending on the context of use, AugmenTable is able to display various interactive touch-enabled controls (e.g., play or pause a movie, move to next or previous item on a list).
- User posture and position. The force-sensitive resistors and load sensors that are installed in SmartSofa’s back and under its bottom pillows provide information regarding the user’s posture (i.e., user leans back or forward) and position (i.e., middle, left, right) while seated. That way, when interactive controls appear on AugmenTable, they are displayed within the user’s reach area.
- User presence. The force-sensitive resistors and load sensors of the SmartSofa, along with the motion sensor mounted on the ceiling, permit the detection of user presence inside the room. Knowing when one or more users are inside or leaving the room is quite important for deciding when to start or pause specific applications (e.g., turn on the TV when someone is in the living room, pause the movie when a user leaves the living room, etc.)
- Object detection. When a physical object is placed on top of AugmenTable, its presence can be identified via sophisticated software. This software cannot identify the type of the object, but it can estimate the space it occupies. That way, the interfaces projected on the coffee table get rearranged in order to display the available information in areas that do not get hidden by the identified object(s).
- Remote control. A three-dimensional gyroscopic remote control can be used as a mouse or keyboard. In its front side, it includes on/off buttons, navigation arrows, and arithmetic controls. Its back side includes a keyboard that enables text input.
- Voice recognition. Simple voice commands are supported (e.g., play, pause, stop), while the users can also record short phrases as vocal messages.

The “Intelligent Living Room” currently uses the aforementioned multimodal methods to monitor the interaction of the users and translate them, in a context-dependent manner, into commands for the developed ambient applications. In principle, all the modalities are available across the entire application spectrum; nevertheless, in certain cases, they become temporarily disabled to improve interaction. For instance, voice input is disabled when TV or Music is playing in order to avoid false positives, as the audio output could trigger the voice recognition component. 

## 5. UX Evaluation

Evaluating an intelligent room that includes many diverse artefacts and employs various ambient applications and interaction modalities is a complicated task. Considering that the living room is part of a larger space (i.e., the “Intelligent Home”), the overall user experience of living in the intelligent house and sharing it with other people (family or friends) should be taken into consideration. Additionally, the context-of-use can be anything that falls within the realm of everyday life activities for any of the involved parties (i.e., users, residents), which resembles the “anytime/anywhere” paradigm that emerged with the proliferation of mobile devices referring to equivalent scenarios of mobile use [162]. 

However, experiences that occur within such spaces can be evaluated to assess not only targeted scenarios and use cases, but also the overall experience with environment. To that end, a series of user-based evaluation experiments have been conducted—while others are in progress—in order to (i) assess the functionality of the ambient applications, identify any unsupported features and uncover severe usability errors, and (ii) draw insights and assess the User Experience (UX) with the Intelligent Living Room by observing the users interacting with the various artefacts (e.g., SurroundWall, AugmenTable), end-user applications (e.g., AmITV, CaLmi), room-wide services (e.g., user monitoring), and the environment as a whole.

### 5.1. Formative User-Based Evaluation of AmITV and AugmenTable

A small-scale formative user-based evaluation experiment was conducted in order to assess the user experience and the interaction paradigm used by—older versions of—AmITV and AugmenTable (as described in [126]). A total of five (5) users of ages 25–40 years participated in the experiment and interacted with the two artefacts, while engaging with some of the developed ambient applications (i.e., entertainment applications, notifications, and the communication application). According to Nielsen [163], testing a system with five (5) users permits the detection of approximately 85% of the problems in an interface, increasing the benefit–cost ratio. Hence, despite the small number of participants, the formative evaluation experiment provided valuable insights, which led to the updated versions of AmITV and AugmenTable and of the accompanying ambient applications, reported in Section 4.1.2, Section 4.1.3 and Section 4.3 respectively.

Overall, the users expressed positive comments regarding the systems, and they particularly praised the fact that they were easy to use, responsive and intelligent, while they found the experience “pleasant” and “fun”. The most important issues revealed were related to the available interaction modalities. In more detail, in the previous version of AmITV, the Kinect device was mounted on top of the TV, permitting the users to perform hand gestures (i.e., waving, sweeping right/left and up/down, and zooming in/out) in order to navigate through the available UI elements or control the application that was in the foreground (e.g., volume up/down or play/stop when a media player was active, zoom in/out or previous/next when the photo viewer was active). However, this type of interaction was proven ineffective, since the users had to make long, repetitive, and physical wearing movements with their arms. To this end, in the newest version of *AmITV*, the Kinect device is used solely to monitor the presence of people and track their movements, while mid-air subtle gestures are still available through the Leap Motion sensors that are embedded in the SmartSofa’s side arms. That way, the users can perform gestures by slightly moving their hand, while resting their arm on the sofa. 

On the other hand, the coffee table originally described in [126] has been completely redesigned following evaluation. The original version featured physical sensors embedded in the furniture, which not only limited the physical space that could be used for placing objects (such as coffee mugs, tea cups, plates, books, etc.) on the table’s surface, but also caused increased discomfort when users had to stretch in order to reach them. To this end, the new AugmenTable consists of a plain white wooden table that hosts a projector above it. This new setup permits the “dynamic” projection of the available UI controls near the position of the user so as to always be within reach; the exact location is determined using force-sensitive resistors and load sensors embedded in SmartSofa that detect where the user is currently sitting. Furthermore, through sophisticated software, the system can identify whether an object is placed on top of the table and appropriately rearrange the projected interface so that the available information does not get occluded. 

Finally, apart from the aforementioned issues, some minor usability issues were also identified regarding the user interface of the ambient applications (e.g., inconsistencies on the placement of some buttons, unclear icons, not obvious meaning of some labels). These issues were resolved and incorporated into the newest version of the ambient applications described in Section 4.3. 

### 5.2. User-Based Evaluation of ParlAmI

Following a small-scale formative user-based evaluation experiment, which is described in [148], a summative evaluation experiment was planned. After addressing the issues identified in the first experiment, sixteen (16) users of ages 20–45 years were requested to follow a specific scenario in order to create two rules that would dictate the behavior of the intelligent environment. The first rule was relatively “easy”, including a simple trigger and a simple action, so that the user would grasp the “basics” of the rule making, while also having their confidence boosted. The second one was more complicated, since it included multiple actions (e.g., when it is eight o’clock in the morning, I want the alarm to ring, the lights to turn on, the coffee machine to start, and my scheduled to be displayed at the SurroundWall above the TV). 

After analyzing the results of the evaluation, it was revealed that the majority of the participants found the system easy to use, very responsive, and intelligent, while they also admitted that they would use it in their daily life. Half of them expressed a positive opinion toward the custom UI elements that allowed them to select an option instead of typing it, while they also found the use of pictures particularly helpful (Figure 7b). Particularly, 40% of the users revealed that they would enjoy more interactive UI elements, and a custom context-sensitive auto-completion mechanism (e.g., when characters ‘w’ and ‘h’ are typed sequentially, the suggestion could be “when” rather than “where”), so as to reduce the amount of typing. Additionally, 80% of the participants stated that the interaction was natural and that they enjoyed the interactive conversation that made them feel as if they were exchanging messages with a friend. However, 40% of them pointed out that the confirmation mechanism was too tedious, since it was repeating previously acquired information way too often. Nevertheless, participants who were not used to messaging applications, and admitted to “forgetting easily”, were glad to have this feature. This issue can be easily resolved in a following version through preferences that would allow users to set the frequency of confirmation provided (i.e., feedback). Another valuable insight gained through the experiment was that 65% of the participants would prefer to be able to define the complete rule in a single utterance (i.e., sentence), instead of building it up step by step. 

### 5.3. User-Based Evaluation of CaLmi

A user-based evaluation experiment was conducted in order to identify whether the use of a relaxation program in an intelligent environment has a positive effect on a user’s stress levels. The goal of this experiment was to provide valuable insights regarding the effectiveness of intelligent environments in stress management. In more detail, eight (8) users of ages 25–54 years had to perform a specific relaxation program inside the “Intelligent Living Room” at a time that they felt stressed. At first, each user was asked to wear the Empatica E4 wristband (that collects various physiological signals) for two (2) consecutive days, and note down the times of the day at which they thought that there were increases in their physiological signals due to (a) a stressful event, (b) physical exercise, or (c) some other cause. This information was later used in order to determine the range of the physiological signals for each participant and identify the values that are likely to signal high stress levels. Next, in a time period of three (3) working days, each participant made use of CaLmi either on demand or automatically when high stress levels were detected.

The relaxation program selected for the evaluation was “Exposure to Nature”. It was offered through the CaLmi system in two sessions, with different modes per session (multisensory or monosensory mode), and in a random order for each participant. The multisensory session aims at activating (a) the sense of sight by displaying a video of a forest waterfall on the main living room wall and adjusting the color (i.e., it takes a greenish-blue hue of the waterfall’s water) and the intensity (i.e., it decreases) of the room lighting, (b) the sense of hearing by reproducing relaxing music and forest sounds (e.g., running water and birdsong) via the room’s speakers, and (c) the sense of smell by diffusing the lavender scent using the scent diffuser. The monosensory session aims at activating only the sense of sight by displaying the same video of a forest waterfall on a tablet device without sound. The participants continued to wear the Empatica E4 wristband during the sessions and one hour after their completion in order to record their physiological signals and thus possible changes in their stress levels. Furthermore, before and after each session, the participants filled in a questionnaire regarding their perceived stress level. Finally, upon completion of these phases, they were interviewed about their experiences with the system.

The evaluation results confirm that a relaxation session is more effective and satisfying when using the technological equipment and installations of the “Intelligent Living Room” in order to activate different senses (multisensory mode), and is not limited to the visual sense alone (monosensory mode). However, the sample size was small, and further investigation is planned. According to the information provided in the questionnaires, 62% of the participants thought that they were less stressed after the multisensory session in comparison with the monosensory session, while all participants felt calmer, satisfied, sleepy, and pleased after the multisensory one. Moreover, the participants would use CaLmi in their everyday lives in order to receive multisensory, context-aware, personalized interventions for stress reduction. In addition, EDA signals revealed that 62% of the participants were calmer after the multisensory session in comparison to the monosensory session. In more detail, all participants except for one seemed to be more relaxed after the relaxation sessions, and their EDA values were reduced by at least 29% and on average by 49% after the multisensory session, while in the best case, they reached 92%. 

## 6. Results

The work described in this paper is part of an ongoing process that began a few years ago, aiming to equip an entire facility containing simulation spaces (including a two-story apartment that hosts the “Intelligent Home”), with human-centric intelligent artefacts, applications, and services, so as to create fully featured environments that have the ability to accommodate end users (i.e., students, residents, doctors, health practitioners) in an unobtrusive and pleasant manner. The “Intelligent Living Room” has been among the first rooms that became functional and was treated as an initial design and development case study, with a view to acquiring and consolidating design knowledge. To that end, an iterative UCD process has been followed for every intelligent application built in its context, while various tools have been created to assist that process such as UInify, AmITest, and Wizard of AmI. The environment has been populated with an IoT infrastructure, smart objects and furniture, and interoperable home services (i.e., AmIHomeOS), while end-user applications and scripts that define the behavior of the technological facilities (i.e., business logic) have been deployed to create a pervasive, intelligent, and personalized experience. Toward creating a holistic approach, we have also built end-user tools that permit the customization of the intelligent behavior, whereas various sophisticated components satisfy key HCI requirements (i.e., multimodality, consistency, common look and feel). 

Many of the implemented ambient applications were tested during formal evaluation experiments (Section 5), while hundreds of informal demonstrations have been offered to visitors of the “Intelligent Home”. The evaluation experiments in combination with the feedback received during the demonstrations has revealed positive findings, which were very encouraging regarding the future of such an environment. Moreover, the continuous interactions by the development team that works within the intelligent environment and uses its facilities/services on a daily basis, along with the active participation of end users during the entire lifecycle of the design process, aim to ensure practical utility, value, and acceptance. At this point of time, the “Intelligent Living Room” is ready to host in situ evaluation experiments that will assess AmI applications and novel interaction techniques during a longitudinal user-based study, while work is ongoing in the kitchen and the bedroom areas. The “Smart Office”, the “Intelligent Classroom”, and the “AmI Garden” (i.e., a small experimental IoT greenhouse) are three other examples of environments under development.

Our experiences from the endeavor to satisfy the requirements described in Section 3.3 revealed several interesting insights and conclusions. The most important ones that should be taken into consideration when designing and developing intelligent spaces are provided below (Section 6.1, Section 6.2 and Section 6.3). Another aspect that became clear is that the role of the living room shifts from being merely a room for social events and relaxation to a multipurpose technologically augmented home facility (Section 6.4). Table 1 demonstrates how the “Intelligent Living Room” satisfies the requirements described in Section 3.3.

### 6.1. Design Process Insights

#### 6.1.1. Should the Design of an Intelligent Space Follow a Bottom–Up or a Top–Down Approach?

The process of synthesizing an environment by integrating various services, applications, and smart artefacts can be considered similar to “the chicken or the egg” dilemma; should we design with a top–down approach (i.e., a smart home should contain smart rooms, which should contain artefacts/services) or a bottom–up approach (i.e., smart applications/services should be combined to create an intelligent environment)? Can we develop a smart application without designing the smart environment that it will inhabit, or should we first define/design the smart environment and then populate it with applications? Can we actually fully design an environment without designing (at least some of) its components beforehand? In practice, we adopted a combination of the two approaches, which were effectively employed in parallel. The infrastructure and layout of the living room was loosely defined before the applications, but given that the available space was empty (with no furniture), there was the opportunity to conceptualize different alternatives to better fulfill the requirements selected from the previous phases.

#### 6.1.2. Tools that Support Interaction Prototyping in such Complex Environments Are Mandatory

Being able to quickly evaluate interaction techniques and system behavior early in the design process is of utmost importance. This process was proven to be cumbersome inside an intelligent environment where the overall design did not target a few devices only, since different interaction modalities such as speech interaction and air gestures were included as well; to this end, the “Wizard of AmI” has been developed to address that particular shortcoming. Generally, in order to properly assess the User Experience (UX) of intelligent spaces, it is important to equip designers with appropriate prototyping tools to assess the overall ambient experience.

### 6.2. Interaction Design Insights

#### 6.2.1. Use Multiple Screens in an Orderly Manner

Within Ambient Intelligence environments, an abundance of displays of various sorts is available (e.g., TVs, wall/surface projectors, tablets, smartphones, wearables, and smart watches, screens of different sizes embedded in appliances); nevertheless, their excessive use results in an overwhelming and stressful situation that negatively affects the user experience [10], as the users feel disoriented (i.e., they do not know in which screen to focus their attention) and cannot deal with the information overload (i.e., they get bombarded with information from every corner of the room). Therefore, designers should: (i) minimize screen abuse (even in screensaver mode) and deactivate any unnecessary displays when not needed, (ii) apply the principles of minimalism and refrain from the tendency to use all the available displays to present information only for the sake of doing so, and (iii) assist users by presenting information on the screen(s) that they would probably seek for themselves (e.g., live feed from the security camera on the coffee table when watching TV).

#### 6.2.2. Avoid Duplication of Information

Another common pitfall is that designers while trying to maximize the use of the available screen real estate, tend to include small information widgets (e.g., clock, weather) to “fill-in” regions that would otherwise remain empty. Doing so for multiple displays that co-exist in the same physical space would result in displaying the same information in multiple locations for no apparent reason. An analogy from the domain of web design is that websites have removed the clock from their homepage, since most users already have a clock on their (computer) screen. Therefore, designers should try to avoid any unnecessary duplication of information since it might overwhelm the user; the only exception would be information that is critical to receive, in which case duplication might ensure that.

#### 6.2.3. Pay Particular Attention to the 3Cs

With the new paradigm of ecosystems of devices that can all host the same service or functionality (e.g., viewing a video on the TV or the smartphone), a new design guideline was identified: pay attention to the 3Cs (Section 4.4.1): consistency, convergence, and continuity. *Consistency* refers to the common look and feel and (interaction) design patterns throughout the ecosystem of devices, *convergence* refers to the seamless transition of the user experience from one device to another when using a service, which allows the experiences to have *continuity* (switching devices and picking up from where you left without problems). The same principle regarding ecosystems of devices is also true for the intelligent environment. This is natural, since the intelligent environment (and specifically the intelligent living room discussed here) is in fact an ecosystem of devices. Even more so, since the intelligent environment is conceptually based on an even stronger metaphor of a single entity than a service that spans devices; services are housed in a single physical environment (room), which needs an even stronger sense of identity (the look and feel) and strong continuity to allow for a seamless, natural User Experience).

#### 6.2.4. Enable User Interface Compositions

During the course of prototyping and evaluation, it was soon evident that there will be a need for literally dozens of user interfaces and variations of them (depending on context and user) across different devices. Sometimes, predicting the need for UIs right from the start is almost impossible since ideas become refined and worked upon during development, and new devices or circumstances of use come into play. The solution to this problem of high demand for UIs and the uncertainty factor of when and where to use them has led to the idea of producing real-time front-end user interfaces on demand. More specifically, users should be empowered to compose flexible UIs by creating “mashups” that combine data and services from existing interfaces, resulting in personalized UIs. UInify, which is still a work in progress, supports the Intelligent Living Room on that matter, ensuring design and UX consistency among devices by applying a common style guide while additionally enabling users to compose their own rules that can guide automatic user interface composition based on various run-time parameters (e.g., screen size, available interaction modalities, context of use).

#### 6.2.5. Personalize Information to the User

One of the most desirable requirements of intelligent environments is a highly personalized user experience that is customized to the user’s preferences and habits. In our case, this meant carefully going through each screen utilized in the living room to determine the type of information available to each user, thus providing a spatial distribution of information according to the location of each user in the room, as well as filtering the information that is publicly viewable by all. Certain pieces of information would show up during testing (such as personal notifications), and it was clear that even previews of notifications might be sensitive for all to see. The obvious solution to the problem was blocking sensitive information from being displayed when other people are in the room. A more thorough treatment of the problem would be to allow for each user to set up their own privacy settings for every device and service available in the environment.

#### 6.2.6. Minimize the Number of Notifications

An intelligent environment, especially one that is a part of an even larger environment (as the Intelligent Living Room is part of an Intelligent Home) will feature many services running in parallel. It was quickly obvious that a great number of notifications are produced, and without any prioritization and user-defined preferences, the result is overwhelming and rather annoying, too. Critical notifications may get buried in the noise. Not everything needs to be presented to the user. Certain information is quite trivial, and other information might be too sensitive and private (see Section 6.2.5). Therefore, it was determined that very few notifications should be pushed; instead, the software ought to provide a sort of notification board for each user to explicitly browse that would contain all the notifications and a filtering mechanism to manage the high amount of information available.

#### 6.2.7. Draw Attention to Displayed Information When Needed

Some notifications as noted may be important or even critical (such as safety notifications). Since a great number of notifications are produced by the environment, and a tendency emerges to ignore them after a while, it was deemed necessary to “boost” specific categories of notifications (such as safety critical notifications or emergency messages from trusted users) both visually and aurally. 

#### 6.2.8. Employ System-Wide Natural Language Interaction

Users enjoy natural language interaction, and the vision of interacting with a computer system or intelligent environment naturally through speech is perhaps the holy grail of human–computer interaction. Significant progress has been made, and speech recognition systems that are widely available have become very accurate. However, the system must be fast, accurate, and able to understand non-native speakers as well. So far, the level of accuracy for Greek speech recognition is not the same as for English. The same applies to speech synthesis; users appreciate natural language feedback; however, the lack of correct intonation makes speech flat and hard to decipher.

#### 6.2.9. Cater for Simultaneous, Context-Sensitive, Multi-User Interaction

An ongoing challenge is managing the problem of having multiple users addressing the system simultaneously. This presents a challenge in terms of deciphering and interpreting multiple voices/input at once, but also managing conflicts that might emerge between what the users are asking the system to do. The latter is a challenge that applies to all available modalities in the intelligent room. The personalization of information mentioned in Section 6.2.5 is part of the solution, since it can be expanded to include the user interface and the options made available to users. For example, the media player system displays the video controls on the AugmenTable; a single instance of them is to not allow for two users to concurrently manage the playback of a video (which would be rife with conflict and would not make sense), but the house resident with the highest hierarchy at any given time (depending on context; it could be the person who turned the system on, the most senior resident present, etc.) should have the playback controls available on their mobile device controlling the application.

#### 6.2.10. Use Body and Hand Gestures with Caution

Gestures that require users to raise their arms and move them for a long period of time for frequent and repetitive interactions should be avoided, since they can quickly become tiring. On the contrary, mid-air hand gestures are a better alternative when used in moderation. Moreover, a common gesture vocabulary should be used consistently across all applications; e.g., swipe left to view the next item of a list (e.g., photograph, security camera feed).

### 6.3. Insights about Ambient Intelligence in General

#### 6.3.1. Act Proactively, but Always Let the Users Retain Control

Ambient Intelligence by definition refers to systems that can anticipate user needs and take appropriate actions to support a user’s primary task or related tasks autonomously. Especially in the context of the “Intelligent Home” where the environment unobtrusively monitors the users and their activities, the fact that it can implicitly predict their desires and automatically act on their behalf on simple matters such as adaptation of the room’s atmosphere (lights, temperature, aroma, music volume, etc.) or execution of commands (e.g., pause a movie when the user moves away from the sofa) pleases the users. However, as the majority of them expects, the system should wait for an approval before making any major changes, at least for the first time. Moreover, even if an action gets initially approved, the users should always be able to override system decisions and customize at run time every aspect of the environment according to their preferences.

#### 6.3.2. Immersive Experience Positively Affects User Satisfaction

From our experience, users particularly enjoyed the sense of immersion offered by the SurroundWall artefact, while some of them suggested extending the projection area to the adjacent available walls. Therefore, when possible, depending on the task’s nature, consider altering the overall environment (e.g., adjust light color and intensity, enhance wall surroundings via projections, emit scents, play relevant sounds/music) to create an immersive experience that can positively affect user satisfaction. 

#### 6.3.3. Inherently Support Multi-User Interaction, Customization, and Cater for Privacy

Domestic environments usually host multiple users, who should be expected to simultaneously interact with their facilities. Therefore, such intelligent environments should on the one hand let the users easily customize the behavior of the room according to their needs and preferences, and on the other hand anticipate and—whenever possible—seamlessly resolve conflicts and race conditions. For instance, when the user who is about to enter the house wants the light on, while the user who has fallen asleep on the couch wants it to remain off, the system should automatically turn the light on, but dim its intensity to accommodate both needs. Moreover, contextual information and prioritization can be used to ensure that the correct action will be taken at any given moment; e.g., prevent children from unlocking the main entrance using a voice command or guests from peeking through the home’s surveillance cameras. Following the same line of thought, personal data should be kept away from the prying eyes of those not authorized to see them. In particular, the system should take into consideration each user’s privacy settings when multiple users are simultaneously present, to decide (i) whether personal content (e.g., notifications, messages, photos) should become available or remain hidden, (ii) where it should be delivered (e.g., smartphone, speaker, TV, smartwatch), and (iii) how it should be represented, depending on the current context of use (e.g., a short notification box saying “You’ve got a new e-mail from a co-worker” or a notification popup saying “John sent you an email entitled ‘Budget estimation for project X’” along with a short preview of the email’s body). Finally, given the enormous amount of data that an intelligent home is able to collect about its residents, appropriate security mechanisms should be provided to keep such data safe and limit their exposure to external services without the users’ consent.

#### 6.3.4. Support End-User Development via a Versatile System Architecture

An AmI environment is powered with many different technologies that co-exist and cooperate in order to enhance the surroundings and proactively and intelligently react to human needs. Therefore, better horizontal integration between application layer protocols is necessary to ensure that the respective AmI components and applications can effectively interoperate while remaining loosely coupled to ensure scalability and maximize reusability. In the context of the Intelligent Living Room, the AmI-Solertis framework (Section 4.2.1) is used to enable developers to realize AmI scenarios through a scalable tooling infrastructure that supports: (i) the exploration of the available intelligent facilities (e.g., technologically augmented artefacts, computational resources, SaaS), (ii) the compilation of the desired intelligent behavior (i.e., business logic) through versatile authoring tools (e.g., source code editor, graphical editor, virtual reality or VR) into independent units of software (i.e., AmI scripts), (iii) the provision of detailed real-time monitoring of the intelligent infrastructure, and (iv) the facilitation of live logic modification at run time (e.g., remote calls interception, service replication, dynamic reconfiguration) to adapt the environment to the needs of the user and improve the overall Quality of Service (QoS). In general, the emerging paradigm of End-User Development (EUD) should be supported so as to empower users to explore the initial system’s functionality, adapt software to their personal needs, or develop new innovative applications.

#### 6.3.5. Rely on Industry Standards to Enable Synchronous and Asynchronous Intercommunication

The communication middleware of an intelligent environment should: (i) be real time, scalable, and cross-platform, (ii) rely on well-established technologies, (iii) support synchronous, asynchronous, and event-based communication, (iv) facilitate resource discovery, management, and updates, (v) enable service composition and self-exposition, (vi) simplify service updates and deployment, (vii) streamline the introduction of existing external services, (viii) be developer-friendly, and finally (ix) allow the introduction of new components that extend its functionality. Currently, Hypertext Transfer Protocol (HTTP) and Representational State Transfer (REST) seem to be the most prevalent technologies that empower communication devices and platforms, while being able to support all types of AmI applications and their desired features (e.g., interoperability, flexibility, extensibility, security, low latency). Unfortunately, REST does not yet accommodate a standardized event mechanism by design. Reflecting on the benefits of both synchronous and event-based communication, the Intelligent Living Room utilizes the Hybrid Communication protocol of the AmI-Solertis framework, which combines the industry standard REST protocol with a custom intermediary message broker to integrate heterogenous services in a standardized—yet agnostic—manner. In principle, the intercommunication infrastructure of an intelligent environment should accommodate both programming paradigms to enable the creation of versatile scenarios of use (i.e., trigger an action on demand or as a response to stimuli).

#### 6.3.6. Increase Acceptance Using Off-The-Shelf Products and Improve Accuracy through Data Fusion

The Internet of Things (IoT) is the concept of a system of interrelated computing devices, mechanical and digital machines, objects, users, and services that can have the ability to transfer data without requiring human-to-human or human-to-computer interaction [164], so as to solve problems in new and more effective ways. The vision behind this concept is that increased connectivity will facilitate automation, visibility, and access to services, which subsequently will enable companies and governmental organizations to tailor products and services to individual needs and ensure that they are delivered accurately and effectively. Smart objects along with their functionality constitute domain-specific applications (vertical markets) targeting a wide spectrum of spaces, such as consumer/domestic, commercial, industrial, agricultural, medical, transportation, and so on, while ubiquitous computing and analytical services form application domain-independent services (horizontal markets). The increased interest in the domain of IoT is apparent in the massive growth of the respective market, which is expected to grow from an installed base of 15.4 billion devices in 2015 to 30.7 billion devices in 2020 and 75.4 billion devices in 2025. 

Driven by the growing number of physical objects connected to the internet, which can see, hear, think, and perform functions, share information, and coordinate decisions, an intelligent environment should be able to integrate any kind of smart object and formally expose their functionality (e.g., OpenAPI Specification) through service-specific wrappers, in order to simplify their utilization. Such an approach guarantees that end users can “bring their own devices”, rather than having to buy additional, vendor-specific hardware to make their environment intelligent, which in turn will minimize their expenses and can potentially increase the acceptance rate. 

Finally, since an abundance of smart objects is expected to be found in intelligent environments, they should permit the aggregation and fusion of related contexts by multiple sensors (i.e., synergy) or at different time instants by a single sensor, so as to improve the quality of the information output (e.g., accuracy, certainty, completeness). Principally, employing more than one sensor can enhance the synergistic effect in several ways: increased spatial and temporal coverage, increased robustness to sensor and algorithmic failures, better noise suppression, increased estimation accuracy, and the introduction of new information to the current knowledge that can allow for a more complete view of the world.

### 6.4. The New Roles of the Living Room in the Emerging Technological Era

Integrating Ambient Intelligence technologies into a traditional living room transforms it into an “Intelligent Living Room” constituting a smart ecosystem that aims to: (a) enhance leisure activities by providing a rich suite of entertainment applications, (b) implement a home control middleware, (c) act as an intervention host that is able to contribute in illustrating appropriate content when the users need help or support, (d) behave as an intelligent agent that communicates with the users in a natural manner and assists them throughout their daily activities, (e) present a notification hub that provides personalized alerts according to contextual information, and (f) become an intermediary communication center for the occupants (e.g., family). The following subsections undertake to describe the aforementioned functionality.

#### 6.4.1. The Living Room as an Entertainment Center

Entertainment is undoubtedly an integral part of human life; it is a form of activity that holds the attention and interest of an audience or gives pleasure and delight. To this end, entertaining people was one of the primary goals of televisions. Indeed, nowadays, TVs permit users to watch movies and shows, play games, browse pictures, listen to music, surf the internet, etc., either alone or with the company of friends and family. Introducing new technologically enhanced artefacts in the living room, such as secondary displays (i.e., AugmenTable and SurroundWall) and multimodal input sources (e.g., SmartSofa, AugmenTable, User Tracking service) permits inhabitants to have a better User Experience. That is because for each of the aforementioned activities, the users of the “Intelligent Living Room” have direct access to supplementary content and novel interaction techniques. 

#### 6.4.2. The Living Room as a Control Center

In the case of domestic life, the advancement of IoT [165] in combination with cloud computing [19] has led to an abundance of web-enabled devices and services for smart homes [48]. Given that in such environments a great amount and variety of devices and services exist, it is important that users can monitor and control them through a simple unified environment. Additionally, since many daily activities are linked to the living room—meaning that residents spend a lot of time inside that room—it is apparent that giving the users the opportunity to control their entire house from the comfort of their sofa is highly desirable. To this end, the “Intelligent Living Room”, taking advantage of the services offered via AmIHomeOS and employing the Home Control application (Section 4.3.4), is transformed into a control center that allows the management of any web-enabled device and service of the house.

#### 6.4.3. The Living Room as an Intervention Host

As already mentioned, a TV is the most used I/O device inside a home setting, hence being the most appropriate channel for communicating with the inhabitants when necessary. This is why the TV as well as the secondary displays of the “Intelligent Living Room” are suitable intervention hosts for LECTOR. As soon as LECTOR identifies a user behavior that requires an intervention, it initiates an exploratory process to identify the most suitable intervention and the proper artefact for hosting it. Each of the developed ambient applications (Section 4.3) can be used as channels to present LECTOR’s interventions, and can be initiated on demand (via their exposed REST API) with specific content, so as to help or support the users in need. In order for an application to be part of an intervention, it is required to conform to AmI-Solertis SaaS specifications, ensuring that it will be able to receive and execute LECTOR’s commands. 

Regarding the available intervention hosts of the “Intelligent Living Room”, they can be categorized as follows: (i) displays (i.e., AmITV, AugmenTable, and SurroundWall) that can present video, pictures, and text, (ii) speakers and sound outputs, (iii) various devices and appliances (e.g., lights, locks, blinds, scent diffuser) that can be controlled either via their own Application Programming Interface (API) or using dedicated solutions to that matter.

CaLmi (Section 4.3.5) is the first integrated approach for turning the entire “Intelligent Living Room” into an intervention host. However, there are many other cases in which the living room can act as an intervention host and assist the users in need. For example, consider a user who has been sitting on the sofa watching movies for a quite long time, and the “Intelligent Living Room” displaying a notification on the TV suggesting a short walk.

#### 6.4.4. The Living Room as an Intelligent Agent

The fusion of conversational interfaces into Intelligent Environments enables the provision of more intuitive interaction paradigms (i.e., natural language dialogues) between users and intelligent virtual agents (i.e., technological artefacts). The “Intelligent Living Room” behaves as an intelligent agent in the form of a chatbot (Section 4.3.6) that communicates with the users in a natural manner and assists them throughout their daily activities (e.g., suggest home automation scenario based on user monitoring, provides multimedia content recommendations, stress reduction commands, or feedback for user actions). The underlying concept is that instead of communicating with a computer on its own terms by clicking on icons and entering syntax-specific commands, the user can naturally interact with the environment by just telling it what to do, which also makes functions and commands immediately available, without the need to navigate in a menu.

#### 6.4.5. The Living Room as a Notification Medium

Nowadays, inside “intelligent” environments, people are connected to an abundance of web-enabled services. Each of these services has the ability to notify the users of either interesting or urgent events. Inside the “Intelligent Home”, an inhabitant receives various notifications such as medicine reminders, cooking alarms, work-related updates, burglar alarms, etc. Although such notifications are valued by the users, they can also become annoying and distracting. According to [166], in order to avoid overwhelming users with notifications, notification systems need to be able to decide the appropriate time and the best form for presenting them. 

The living room is a social and individual space in which technology plays an important part [9]; many activities are linked to that room, and inhabitants spend considerate time there with their friends and family. These characteristics of the living room make it an excellent candidate for presenting notifications in any of the available displays (i.e., AmITV, AugmenTable, and SurroundWall). 

#### 6.4.6. The Living Room as a Family Communication Hub

In today’s evolving world, people are consistently seeking to better balance their professional and personal life. Amongst others, people attempt to balance this equilibrium using technology in their day-to-day activities (e.g., sharing of photos and calendar information between family members) [167]. Without doubt, effective communication within the family can lead to better relationships between its members [63]. The use of internet and the various communication technologies (e.g., email, instant messaging, social networks) broadened the ways that people can stay in touch, maintain family contact, and communicate with people with whom they normally would not have the chance [168]. Through taking advantage of the environment’s “intelligent” facilities and employing the ambient communication application (Section 4.3.3), the “Intelligent Living Room” provides a multimodal and ubiquitous communication hub that addresses the need for family communication. 

## 7. Discussion

The emergence of the Ambient Intelligence (AmI) paradigm and Internet of Things (IoT) devices and services unveiled new potentials for the domain of domestic living by shaping the “Intelligent Living Room” along with an ecosystem of smart services built around it (e.g., entertainment applications, middleware, virtual agents). Being one of the “busiest” rooms in a domestic environment, where diverse daily activities take place with the participation of many users simultaneously, the living room can be considered as the perfect candidate to showcase the issues and challenges that emerge in similar environments during their technologically oriented transformation into “Intelligent” spaces. This paper reports our experiences from the endeavor to develop the “Intelligent Living Room” located in the Ambient Intelligence Facility at FORTH-ICS. In particular, it provides insights into how the “Intelligent Living Room” (i) realizes its newly emerged roles and its promising potentials at the dawn of Ambient Intelligence, (ii) presents the process that was followed in order to design the living room environment, (iii) introduces the hardware and software facilities developed aiming to improve the quality of life of the residents of such a space, and (iv) reports the findings of a series of user-based evaluation experiments that assessed the functionality and utility of the ambient applications and their overall User Experience.

The complexity of the task is enormous, considering the diversity of the available technologies and the user groups involved. There is an exponential growth in complexity compared to designing for a single device or even an ecosystem of devices. Our approach was based on the design-thinking methodology. This includes the ideation process, which in this work explicitly considered the bigger picture (i.e., the home, the services, and the available technology), the filtering process that included experts from different disciplines, and the prototyping phase that was successfully completed with the help of “The Wizard of AmI”. Additionally, software developers working on the development and integration of the various services (e.g., AmIHomeOS) were present and involved both in the ideation and the idea-filtering process in order to ensure that any emerging needs or dependencies between services were addressed. To the same end, all design concepts for the “Intelligent Home” (including the “Intelligent Living Room”) include a section that lists all the services and applications that are related to the concept.

There is still a lot of work to be done, and there are several aspects that merit further research in the context of “intelligent” domestic environments. First of all, the design and development methodology applied is going to be refined as work progresses and experience matures, with the ultimate goal being to lay the ground for a solid design framework that extends beyond a single room and can be effectively used for reshaping other “traditional” spaces into “intelligent” ones or designing an intelligent environment from scratch. The challenge in this case is not only the increased complexity of the design, but also the incorporation of the notion that the room environment is part of a larger environment (the home, or perhaps the hospital, the school, or the office) with shared services and resources. Moreover, given that a large number of services and ambient applications are actively under development (in the same and other domains) and are going to be introduced in the ecosystem (e.g., smart greenhouse management tools, applications for the “Intelligent Classroom”, a “cooking coach” for the “Intelligent Kitchen”), new scenarios will explore whether the identified roles should be further expanded.

Additionally, from a human–computer interaction perspective, multiple full-scale user-based evaluation experiments are going to be conducted in situ to gather feedback regarding our development so far. Besides the “regular” UX insights, there is great interest to gain insights regarding (i) the optimal combination of modalities per case and (ii) the balance between the space implicitly reacting to the residents’ needs and it becoming a burden or behaving in an offensively condescending manner (i.e., patronizing) toward its residents. Simply put, the users should not be forced to serve the new technologies by doing more work than they did without them, such as setting up a myriad of preferences or teaching the house explicitly what they want or need. Similarly, a house that, for example, automatically starts playing mellow music to calm a user down, based on wearable readings of stress, but really seemingly out of the blue may easily produce the opposite effect. AmIHomeOS will be further enhanced as well. The User Interface development kit for Ambient Intelligence environments (AmI-UI) and the simulator will be extended to support fluid interfaces, which will be generated on the fly and distributed across multiple devices. Finally, the AI components that control the intelligent behavior of the Ambient Intelligence (AmI) Home will incorporate machine learning algorithms and enable dynamic user-oriented configurations.

## Figures and Tables

**Table 1:** Requirements fulfilment matrix.

Requirement	Status	Description
REQ1	√	LECTOR framework
REQ2	√	AmIHomeOS and Ambient applications
REQ3	√	AmIHomeOS and Home Control application
REQ4	√	Entertainment and Communication applications
REQ5–REQ7	~	*Appropriate ambient applications are currently designed*
REQ8	√	Entertainment applications
REQ9	√	Notification mechanism
REQ10	√	AmIHomeOS and Home Control application
REQ11	√	CaLmi
REQ12	√	LECTOR and Ambient Applications
REQ13	~	*Under development*
REQ14	~	*Appropriate ambient applications are currently designed*
REQ15	√	Home Control application (Section 4.3.4)
REQ16	√	LECTOR
REQ17	√	AmI-Solertis, LECTORstudio, and ParlAmI (Section 4.2.2)
REQ18	√	AmI-Solertis, LECTOR, AmIHomeOS
REQ19	√	LECTOR and Home Control application
REQ20	√	AmIHomeOS
REQ21	~	*Under development*
REQ22	√	AmI-Solertis, AmIHomeOS
REQ23	√	AmIHomeOS
REQ24–REQ27	√	Multimodal
REQ28	√	SurroundWall artefact
REQ29	√	AugmenTable artefact
REQ30	√	UInify
REQ31–REQ33	√	Considered throughout the design process
